# Are Complementary Feeding Practices Aligned with Current Recommendations? A Narrative Review

**DOI:** 10.3390/children10050794

**Published:** 2023-04-28

**Authors:** Audrey Nantel, Véronique Gingras

**Affiliations:** 1 Department of Nutrition, Université de Montréal, Montreal, QC H3T 1A8, Canada; audrey.nantel.1@umontreal.ca; 2 Research Center of the Sainte-Justine University Hospital, Montreal, QC H3T 1C5, Canada

**Keywords:** complementary feeding, timing, food choices, parental feeding practices, solid food, infancy, determinants, baby-led weaning

## Abstract

The complementary feeding introduction period (introduction of solid foods alongside breastmilk or formula) is defining in children’s health; however, it appears that many parents do not follow complementary feeding guidelines. Our aim was to describe current parental feeding practices during complementary feeding in relation to current recommendations and explore determinants of adherence to guidelines. We included any relevant studies published within the last decade in French or English and summarized findings by recommendation category. The timing of complementary food introduction varied widely across and within continents (earlier in North America and often delayed in Asia). The introduction of allergenic foods tended to be delayed globally. Although some parents now begin complementary feeding with solid foods (i.e., baby-led weaning), delayed introduction of lumpy textures was still prevalent in the United States and in Europe. The consumption of iron-rich foods was predominantly low in Africa. Added sugars were globally introduced early, especially in America. Evidence for the prevalence of responsive feeding practices among parents is unclear due to the small number of studies. Determinants of complementary feeding practices included parental characteristics, such as age, education, socio-economic status, and race/ethnicity. Interventions aiming to increase adherence to complementary feeding guidelines must account for parental characteristics.

## 1. Introduction

There is compelling evidence suggesting that the first thousand days of life, from conception to around 24 months of age, are decisive in children’s development, influencing both psychological and physiological health [1,2]. The complementary feeding (CF) period is set during this specific period of life and involves the introduction of complementary foods in children’s diet after being exclusively breastfed or formula-fed [3]. The World Health Organization (WHO) and the United Nations Children’s Fund (UNICEF) have identified breastfeeding and adequate CF as the most significant practices in the first two years of life for promoting children’s health [4]. In 2003, the WHO published guidelines for CF introduction which focus on decreasing the risk of nutritional inadequacies and promoting adequate development and health [3]. Briefly, it is recommended to introduce CF at 6 months of age alongside continued breastfeeding (or formula use for mothers unable to breastfeed), to proceed with a gradual progression of food textures and consistencies from 6 to 12 months, to avoid low-nutrient foods and beverages and to limit fruit juices, to offer iron-rich foods daily and iron-fortified CF as needed, and to apply responsive feeding practices [3]. Recommendations regarding CF introduction are rather consistent worldwide, although in Europe, the European Society of Paediatric Gastroenterology, Hepatology and Nutrition (ESPGHAN) guidelines indicate that CF can be introduced between 4 and 6 months [5]. These guidelines are the only ones to specifically mention not to delay CF introduction past 7 months of age in addition to not introducing it too early (<4 months) [5]. Other organizations have also published more recent guidelines, notably regarding the introduction of allergenic foods (to be introduced at the beginning of CF) and added sugars (to be avoided for the first two years) [5,6,7]. Nonetheless, it appears that many parents do not comply with international recommendations regarding CF practices [5]. A better understanding of parental adherence to guidelines and of parental characteristics associated with non-adherence is essential to develop effective early-life interventions targeting CF. In this narrative review, we aimed to describe current parental practices during the CF period and examine whether they align with international recommendations. We also explored determinants of adherence to guidelines.

## 2. Materials and Methods

Our search focused on observational studies published within the last 10 years (2012–2022), written in English or French. We conducted our search from June to August 2022 in the PubMed and Science Direct databases using keywords related to complementary feeding practices, such as complementary feeding, complementary food, solid food introduction, infant and toddler feeding, parent or caregiver feeding practices, and baby-led weaning. Other references, such as official government documents and systematic reviews, were then included until February 2023. We included any relevant studies in humans from all available countries and excluded studies conducted before 2012 and those performed among twins, preterm infants, or among infants and toddlers with serious health conditions which could impact feeding practices. After the identification of eligible studies, we analyzed and classified data according to their corresponding complementary feeding practices, based on international organizations recommendations, mainly those of the WHO. We therefore present a narrative synthesis of findings by recommendation category (timing of CF introduction; introduction of allergenic foods; progression of food consistency and textures, including a brief discussion on baby-led weaning; nutrient content; and responsive feeding practices).

## 3. Results

### 3.1. Timing of CF Introduction

The WHO recommends to introduce solid foods at the age of 6 months, alongside breastmilk or infant formula, following the infant’s readiness cues [3]. Recommendations vary slightly in Europe as mentioned above, where CF introduction is recommended between 4 and 6 months, with an emphasis on the importance of waiting until at least 4 months [5]. A very early introduction (<4 months) of CF may increase the risk of childhood obesity [8] and feeding difficulties [9], while the omission to introduce solid foods at an age-appropriate time may increase the risk of nutritional inadequacies [3,5].

Table 1 summarizes the timing of CF introduction within and across continents, as well as parental characteristics and practices associated with an early introduction to CF. An extensive version of this table (Table S1) is also available in the Supplementary Materials. In the United States, one-third of infants were introduced to CF prior to 4 months according to the 2016–2018 National Survey of Children’s Health [10], similarly to what was also found in Quebec, Canada [11]. A high prevalence of early CF was also found in Brazil [12,13] and Mexico [14] among families of low socioeconomic status and/or low–middle education levels. In Chile, studies conducted among highly educated mothers showed high adherence to the WHO recommendation regarding timing of CF [15,16]. Very early CF was generally less prevalent in Europe [17,18,19,20,21,22,23,24,25,26]; the Toy-Box study conducted across six European countries showed that 1–18% of parents introduced CF < 4 months of age [27]. The majority of parents in these countries introduced CF between 4 and 6 months, which is consistent with European guidelines [27]. Moreover, the 2017 national Scottish maternal and infant nutrition survey showed that only 3% of infants were introduced CF < 4 months [28]. Nonetheless, some European studies have also shown a relatively higher prevalence of very early CF in France [29], the United Kingdom (UK) [30], and Poland [31], similar to that found in North America. In Australia, a recent national survey (OzFITS 2021) showed that the median age for CF introduction was 5 months of age, and only 1.5% of parents began CF < 4 months [32]. In Asia, most parents introduced CF at or after the recommended age [33,34,35,36,37,38]. In China, 87% of parents from the Jiaxing Birth Cohort (*n* = 18 446) introduced CF > 6 months of age [39]. In India, 10% of infants from the Santal tribe had not yet been introduced to CF at 9 months [34]. Only 1–8% of parents introduced CF before the age of 4 months in China [40], India [34], Lebanon [41], and the United Arab Emirates [42]. In Ethiopia, 60–68% of parents introduced CF at the recommended 6 months of age [43,44,45]. Moreover, a large number of studies conducted across all continents suggest that water is commonly offered during the first 6 months of life, for instance in Mexico [14], Brazil [46,47,48], Lebanon [41], China [40], Nepal [49], Sri Lanka [50], and Nigeria [51].

#### Determinants of the Timing of CF Introduction

Several parental characteristics, such as lower maternal education and socio-economic status, younger maternal age, and maternal smoking, were associated with earlier CF introduction (Table 1). These specific parental characteristics may influence CF practices through a lack of knowledge, resulting in a lower adherence to official guidelines [29,81]. These may also contribute to the relationship between higher formula-feeding and earlier CF introduction, since formula-feeding is typically more common among socio-economically disadvantaged parents [56]. It has also been suggested that formula-feeding may lead to reduced appetite regulation mechanisms [82], therefore favoring an earlier introduction to solids. Moreover, Sanjeevi et al. showed that infants who seemed to enjoy milk feeding more (as perceived by their mothers) were introduced to CF later [52]. Conversely, mothers who believed their milk was insufficient to cover their infant’s needs tended to introduce CF earlier than recommended [28,35,43,60,63]. Differences in the timing of CF introduction were also noticeable across continents and cultures. In Australia, immigrant mothers of Asian race/ethnicity introduced solid foods later than those who were born in Australia [73]. Other studies suggest that immigration may in fact alter CF practices through shifts in cultural norms. In a systematic review, Manikam and colleagues showed that mothers of South Asian race/ethnicity residing in industrialized countries introduced CF earlier than their counterparts in South Asia [83]. In the longitudinal Born in Bradford birth cohort study conducted in England, 21% of Pakistani parents introduced CF < 4 months [30], which is uncommon among parents born and living in Pakistan [35]. Some studies have also found associations between maternal occupation/employment, education, and timing of CF introduction. In the Jiaxing Birth Cohort study, mothers who were employed as farmers and less-educated mothers tended to introduce CF earlier than their counterparts [39]. On the other hand, Yu et al. showed that higher maternal education was associated with an earlier introduction to complementary foods in China, possibly through increased workload and obligations leading mothers to return to work shortly after birth [40]. The latter suggests that shorter maternity leave may favor an earlier CF introduction; however, associations between the duration of maternity leave and timing of CF introduction have been inconsistent across studies [73].

### 3.2. Timing of Introduction of Food Allergens (Peanuts and Eggs)

In 2015, Du Toit et al., motivated by the increasing prevalence of peanut allergies worldwide, conducted a randomized controlled trial in which they showed that an earlier introduction of peanuts (between 4 and 11 months) was associated with reduced risks of peanut allergy in high-risk children (i.e., with severe eczema and/or egg allergy) [84]. An early (4–6 months) introduction of eggs into children’s diet has also been associated with a reduced risk of egg allergy [85]. Since then, most organizations recommend to introduce allergenic foods at the beginning of the CF introduction (≥4 or ~6 months depending on guidelines) [5,86,87].

Table 2 presents a summary of the timing of introduction of peanuts and eggs across continents; an extensive version of this table is also provided in the Supplementary Materials (Table S2). In a low-income cohort of mothers from the United States, eggs were introduced on average at ~10 months and peanut butter at ~12 months of age [58]. In this study, formula-fed girls were introduced to peanut butter significantly later than girls who were breastfed, although no significant associations were found in boys [58]. In a Canadian cohort, 21% of infants had not been exposed to eggs in the first year of life and 63% had not been exposed to peanuts [88]. In two cross-sectional studies conducted in Chile and Mexico, egg introduction was delayed > 12 months in most children [16,61]. In Australia, peanuts and eggs were introduced at a median age of 6–7 months, consistent with recommendations [32]. In a Polish study, eggs and peanuts were introduced ~8–12 months [31]. In this study, there was no observed effect of maternal age and education, knowledge in nutrition and predisposition to allergy regarding the timing of eggs introduction; however, lower maternal knowledge in nutrition and maternal education were associated with an introduction to peanuts after 6 months of age [31]. In the UK [28] and in Spain [89], egg consumption was mostly delayed or offered irregularly among infants < 12 months of age. Ben Kayale et al. found cultural differences regarding the timing of peanut exposure, which occurred prior to one year of age in 33% of children in Libya, 45% of Libyan children living in the UK, and only 14% of British children residing the UK [90]. In a South African study, peanuts and eggs were introduced at a median age of 12 months; however, children from urban areas were exposed to these foods much earlier than those living in rural areas [80]. This was especially the case for peanuts, which had not been introduced to 53% of infants in rural areas aged 12–36 months as compared to 6% of children in urban areas [80]. In China, eggs were introduced early during the CF period and were often used as one of the first complementary foods [91]. Similarly, in Thailand, eggs were often the first animal-based protein to be introduced [37]. Yet, this was not consistent across Asia, where the introduction of eggs was often delayed in Malyasia [92], India [93], and Lebanon [41].

### 3.3. Progression of Food Textures and Consistencies

The WHO recommends a gradual introduction of different food textures and consistencies, starting with pureed and mashed foods, and then progressing towards more solid foods so that children can consume family meals by about one year of age [3]. Moreover, some organizations now specifically recommend introducing lumpy textures by 8–10 months [5,6], seeing that delaying the introduction of lumpy foods past 10 months was associated with an increased risk of feeding difficulties, such as pickiness and food refusal [101].

Table 3 presents a summary of the progression of food textures and consistencies during the CF period across continents; an extensive version of this table is also provided in the Supplementary Materials (Table S3). In a longitudinal cohort of low-income mothers from the United States, only 51% of infants were introduced to lumpy textures by 9 months of age [102]. Most parents offered mashed or pureed foods at the beginning of CF, and progressed towards lumpier and solid foods as the child grew older, with 83% of parents still offering some mashed foods at the ages of 11 and 13 months [103]. Pre-masticating was also relatively prevalent (18%) at 9–11 months, especially among parents of African American race/ethnicity [103]. Parents of Hispanic ethnicity also offered purees for a longer duration compared to Non-Hispanic parents [103]. Studies in France also found a prolonged consumption of pureed foods by infants during the CF period [18,64,65]. In a study conducted by Chouraqui et al., only ~50% of infants were introduced to lumpy foods by 11 months [18]. Moreover, Demonteil et al. showed that infants were more readily exposed to textured foods if their parents were younger, introduced CF earlier, and perceived developmental signs of readiness [64]. These parents may have shown less apprehension towards the introduction of harder pieces of foods and began the introduction of textured foods earlier [64]. Other factors associated with increased exposure to a variety of food textures included offering home-made foods and eating with the family [64].

#### Prevalence of Baby-Led Weaning

BLW is a feeding approach that has gained popularity over the past decade, notably in Europe [66,107] and in New Zealand [75]. As opposed to the traditional spoon-feeding (TSF) approach with purees, parents following the BLW method allow their child (≥6 months old) to self-feed various solid foods adapted from the family meals [107]. It is important to note that studies assessing the practice of BLW often use different definitions, hence it is difficult to assess its prevalence. In a cross-sectional study conducted in New Zealand, 18% of parents used BLW (defined as “mostly self-feeding”) for their child at 6–7 months of age, while 11% used a mixed-method approach (50% TSF and 50% BLW) [75]. In the UK, 33% of parents adhered to a strict BLW (self-feeding ≥ 90% of the time) and 17% to a predominant BLW method [67]. The prevalence of BLW (undefined) was lower in a sample of 2999 French mothers, with only 2% of mothers using this method [64]. In Austria and Poland, the prevalence of BLW (self-feeding) ranged from 13 to 19% [106]. Recent studies found that infants fed with BLW were typically breastfed longer and were introduced to CF later [23,69,105]. Parents adhering to the BLW method also typically show higher levels of education [108].

### 3.4. Nutrient Content of Complementary Foods

#### 3.4.1. Introduction of Iron-Rich Foods, Cow’s Milk and Tea

International organizations recommend the introduction of iron-rich foods at the beginning of the CF period and the use of fortified complementary foods or vitamin-mineral supplements as needed to prevent iron-deficiency anemia in infants [3,5]. Moreover, it is recommended to delay the introduction of cow’s milk past 9–12 months, since it is poor in iron and may replace other iron-rich foods [3,5,6]. It is also recommended to delay the introduction of tea, a low-nutrient beverage that can limit the absorption of nonheme iron [3]. Table 4 presents a summary of the introduction of iron-rich foods, cow’s milk, and tea at the beginning of the CF period and their associated factors (extensive version provided in the Supplementary Materials Table S4).

In the United States, recent studies conducted among low-income mothers eligible to the WIC federal program which provides nutritional education and food products, including iron-fortified cereals, have shown relatively adequate consumption of iron-rich foods during infancy [58]. Overall, 82% of parents started CF with iron-fortified cereals [56] and meat products were introduced ~8 months [58]; however, 37% of parents introduced cow’s milk before 12 months (on average ~11 months) [56]. In this cohort, 20% of infants aged 7 months had inadequate iron intakes (<6.9 mg/day), as measured from foods and breastmilk and/or formula intake [103]. Similarly, in Australia, a large proportion of parents started CF with cereals [111]. In Europe, tea was commonly offered at the beginning of the CF period [27] and in France, cow’s milk also seemed to be commonly introduced before 12 months [18,110]. In a cross-sectional study in Spain, meat was introduced at a median age of 7 months and given on average 3–4 days per week from 7 to 18 months of age, whereas eggs and legumes were mostly given 1–2 days per week and introduced later on, but before 12 months of age [89]. In a sample of highly educated mothers in China, 78% had introduced iron-fortified cereals by the age of 6 months [40]. The latest Bangladesh Demographic and Health Survey showed that the consumption of meat, fish and eggs increased significantly over the past decade among children aged 6–23 months, with 67% of children consuming one of these foods during the day of a 24 h dietary recall in 2017–2018 [76]. In addition to the consumption of meat and animal products being relatively low in some countries located in Asia [41,93,97] and in Africa [44,51,100], a high proportion of children in these continents were exposed to tea during the CF period [41,50,79,99]. In Lebanon particularly, 57% of infants were introduced to tea before 4 months [41].

#### 3.4.2. Determinants of the Introduction of Iron-Rich Foods, Cow’s Milk and Tea

Iron-fortified cereals were more regularly offered among parents who used infant formulas [58] and who applied the TSF approach to introduce CF [66,67,69,75]. Conversely, infants who were breastfed [58] and whose parents used the BLW approach [75] were introduced to red meat earlier as a source of iron as opposed to iron-fortified cereals. A study conducted among low-income mothers in the United States showed that iron-fortified cereals and meat were also introduced earlier in parents of Non-Hispanic ethnicity, as well as in parents who were born in the United States [103]. Additionally, mothers who were unmarried and primiparous introduced iron-fortified cereals earlier [103]. Lower income [25,103] and household food insecurity [100] were also associated with a lesser consumption of meat products during the CF period. Among parents who reported being vegetarian or vegan, only few of them excluded animal products from their children’s diet [20,70]; however, they did tend to introduce meat products later than those who reported being omnivore [70]. In Poland, an earlier introduction of cow’s milk was observed among mothers with lower levels of education and lesser knowledge in appropriate CF practices [31], as well as among those following an omnivore diet [70]. Infants who did not present any history of allergies or familial atopies also tended to be introduced to cow’s milk earlier [31]. We did not find studies reporting on determinants of timing of tea introduction.

#### 3.4.3. Introduction of Added Sugars, Juice, and Honey

Most organizations recommend limiting the amount of sugar and salt added to home-prepared meals for infants [3,5]. In the United States, the American Heart Association suggests avoiding added sugars in the first 24 months of life [113], and since 2017, the American Academy of Pediatrics recommends to avoid fruit juices prior to 12 months of age [114]. Moreover, health organizations in the United States and in Canada suggest to avoid honey for the first year of life [6,115], while in Europe it is only recommended to avoid raw honey [5], based on the risk of infant botulism. Table 5 presents a summary of the consumption of added sugars, juice, and honey during the CF period and its associated factors across continents (extensive version available in Supplementary Materials Table S5).

In a study conducted in the United States, added sugars came mainly from yogurts during infancy and from fruit drinks for toddlers [116]. Commercial infant snacks/sweets and sweet bakery products were also major sources of added sugars in young children’s diet [53]. In the WIC ITFPS-2 study, one-third of infants were already introduced to sugar-sweetened beverages by the age of 7 months [102]. Moreover, infants aged 6–11 months in the United States consumed ~1 teaspoon of sugar (4 g) on the day of the recall in the 2011–2016 National Health and Nutrition Examination Surveys [116]. Similarly, in the United Arab Emirates, the daily consumption of free sugar was estimated at ~10 g per day among infants aged 6–11 months [42]. In Brazil, numerous studies have documented a high prevalence of ultra-processed food and juice consumption during the CF period [48,109]. While the proportion of infants exposed to juice < 12 months of age has slightly decreased over the past decades, it still remained at 88% in 2015 [12]. Additionally, a cross-sectional study conducted in Brazil found that 10% of infants were exposed to honey by 12 months [48]. In the large European ToyBox study, fruit juices were introduced at a median age of 6 months, with Bulgarian infants having the earliest introduction [27]. In the French nationwide ELFE Study, the consumption of added sugars increased with age, and 25% of infants consumed meals with added sugars at 10 months of age [123]. In a qualitative study conducted in the UK, Pakistani and Bangladeshi parents mentioned giving ghutti, a traditional remedy used for digestive issues which typically contains honey, during infancy [63]. In Nepal, 52% of children aged 6–23 months consumed foods or drinks with added sweeteners (sugar or honey) on the day of a dietary recall [49]. In Lebanon, sweetened water and juices were introduced at a mean age of ~7 and 9 months of age, respectively [41].

#### 3.4.4. Determinants of Consumption of Added Sugars during the CF Period

In the United States, the consumption of added sugars was found to be lower among Asian toddlers and higher among children of non-Hispanic Black race/ethnicity [116]. Similarly, in a cohort of low-income women, mothers of African American race/ethnicity, who were younger and/or unmarried, introduced pure fruit juice significantly earlier than other groups [56]. In the same cohort, maternal age was negatively associated with child’s age at introduction of sugar-sweetened beverages [56]. Mothers of Hispanic or Latino ethnicity and who were born in the United States introduced sweet foods significantly earlier [56]. Infants of Asian race/ethnicity in Australia were also less exposed to added sugars during infancy as opposed to those of mothers who were born in Australia or the UK [72]. The large consumption of added sugars during infancy in Brazil was especially prevalent among families of low-socioeconomic status and with a lower education level [62,117,121]. Several studies also found an association between formula-feeding and added sugar consumption during the CF period in Brazil [48,109,118]. In Europe, younger maternal age [28,106] and lower socio-economic status [25,28,106] were also found to be predictors of added sugar consumption. In Austria and in Poland, breastfeeding was negatively associated with the consumption of added sugars during the CF period [106]. Conversely, in France, breastfeeding mothers were significantly more prone to add sugars into the meals of their infants [123]. A possible explanation raised by authors is that these mothers tended to prepare more homecooked meals as opposed to purchasing commercially prepared products [123]. In Asia and in Africa, lower maternal education [99,124] and socio-economic status [99] were also associated with a higher consumption of added sugars through processed foods during infancy.

### 3.5. Responsive Feeding

The WHO and other international organizations recommend the practice of responsive feeding with children during CF introduction [3,5,6]. Responsive feeding refers to a special care and attention from parents when feeding their child by, for example, being attentive to hunger and satiety clues, avoiding distractions during meals, being patient and encouraging children during meals without forcing them to eat [3]. In the UK, a study including 60 parents found that 46% of mothers and 26% of fathers adopted a responsive approach, defined in this study as feeding on demand/to appetite [24]. In a qualitative study conducted in rural Sri Lanka (*n* = 18), 84% of mothers did not know about responsive feeding, and none followed a hunger-sensitive feeding schedule [125]. Yet, another study conducted in Sri Lanka found that 87% of mothers interacted positively with their baby during mealtimes, and 84% did not agree that forceful feeding (a non-responsive feeding practice) should be used [50]. Parental pressure during mealtimes is also in direct opposition to responsive feeding and Klerks et al. showed, in a sample of Spanish infants, a higher prevalence of pressure to eat among parents who were younger, worked full time, and who fed their infants commercial foods [89]. A cross-sectional study assessing the CF practices of Thai families (*n* = 108) found that 83% of infants 6–8 months of age did not eat with their family, a proportion that decreased with age, with most infants frequently eating with their family by 18 months of age [37]. An observational study conducted among a subsample of families from the STRONG Kids 2 Study in the United States found that the presence and the involvement of fathers at mealtimes increased responsive feeding practices from mothers [126]. In this study, children were distracted for nearly half of mealtimes by objects or pets, and both parents were also found to be frequently distracted during mealtimes [126]. The use of responsive (or non-responsive) feeding practices has been mostly self-reported in the literature and its practice varies across countries and across socio-demographic groups. Although the evidence is limited to a few studies, mothers may adopt a more responsive approach than fathers, but the presence and involvement of fathers at mealtimes may contribute to increased responsive feeding practices [24,126].

## 4. Discussion

Overall, studies across the world show that many parents do not comply with international guidelines regarding CF introduction. Parental characteristics, such as level of education, socioeconomic status, age, culture, and ethnicity, were associated with adherence to CF guidelines for many recommendations. The feeding mode during infancy (breastfeeding vs. formula-feeding) and weaning approach (BLW vs. TSF) were also predictors of other CF practices.

The high proportion of parents introducing CF < 4 months, especially in the United States (one-third of infants), is problematic considering the previously observed associations between very early CF introduction and increased childhood adiposity and obesity [8]. A longitudinal analysis from a Dutch population-based birth cohort (*n* = 3963) found increased odds of being overweight between 1 and 17 years of age for infants introduced to CF < 4 months compared to ≥4 months of age [127]. Associations of early CF and increased adiposity persist even when considering potential interactions between the duration of breastfeeding and the timing of CF introduction [128,129,130]. Fortunately, in the United States, data from the WIC IFPS-1 and 2 studies have shown an increased prevalence in timely CF introduction over the years in women adhering to the WIC federal program [56], which may lead to positive outcomes in the future. The association of delayed CF introduction and obesity is not consistent across studies; however, delaying the introduction of CF > 7 months may increase the risk of nutritional inadequacies [3,5] and it is thus important to encourage parents not to introduce CF too early, yet not too late either.

We found an overall late introduction of peanuts and eggs as well as lumpy textures, especially in North America and in Europe. In France, only 10% of children were exposed to different food textures before 8 months, the majority consuming pureed foods only. Similarly, in the United States, only 51–63% of infants were introduced to lumpy textures by the ages of 8–9 months. In Canada and in the United States, peanuts specifically were largely introduced past 12 months of age. This may be caused by parental apprehension towards the development of food allergies, as well as recent shifts in guidelines regarding the introduction of allergenic foods [90]. Nonetheless, this is concerning since recent evidence has shown that peanut allergy has increased significantly over recent decades in the United States, reaching 1.8% of adults in 2015–2016 [131]. Moreover, parents may delay the introduction of harder pieces of foods due to a fear of choking [65]. In BLW, as opposed to TSF, lumpy and adapted whole foods are offered at the beginning of CF. Although it has been suggested that BLW may lead to better satiety-responsiveness in children [132], a recent systematic review including eight studies reported indecisive results for the associations of BLW and child’s later risk of obesity [133]. Whether parents choose BLW or TSF, this review highlights the need for strategies to improve adherence to guidelines regarding textures progression and the introduction of allergenic foods.

Although few parents offered meat at the very beginning of the CF period, many introduced iron-fortified cereals early on, especially in industrialized countries. For instance, 54–82% of parents offered iron-fortified cereals as a first complementary food in studies conducted in Lebanon, Spain, China, and the United States. In Africa, where children’s diets were predominantly based of grains, roots, and tubers, the consumption of iron-rich and iron-fortified foods was relatively low during the first two years of life. In a meta-synthesis assessing the CF practices of immigrant mothers in Australia, the affordability of meat products was found to be a facilitating factor in providing iron-rich foods early in infancy [134].

The consumption of added sugars in the first two years of life was prevalent worldwide, especially in the United States and in Brazil, where 13–50% of infants were also exposed to fruit juice during the first 6 months of life. In the United States, 60–93% of infants consumed sweet foods before the age of 12 months, and in Brazil, 79–94% of infants consumed ultra-processed foods during the CF period. This is important considering that sugary processed foods may contribute excessively to daily energy intake and/or replace nutritious foods [3,5,6], especially during infancy where a limited amount of food is consumed daily. Additionally, infancy is a key period in the development of food taste and preferences [135,136], and studies have shown that the consumption of ultra-processed foods during childhood may predispose to childhood obesity and later cardiometabolic diseases [137]. Findings included in this review suggest that guidance for parents remain needed regarding food choices during CF introduction to avoid and limit added sugars.

Responsive feeding was negatively associated with children’s weight status or adiposity in most studies included in a systematic review [138]. It is however difficult to conclude as to the proportion of parents who practice responsive feeding. There are few cohort studies assessing this practice, and most studies relied on self-reported data. Nonetheless, effective interventions to improve responsive feeding practices among both parents appear to be needed. In Australia, an intervention aiming to increase the use of responsive feeding during early infancy showed healthier eating behaviors in children, lasting through to the age of five [139].

Limitations to this narrative review include the different definitions of feeding practices examined and differing methods of assessment across included studies, which hindered our ability to compare data. Some countries were also underrepresented in the literature as compared to others, which is why most continents were not separated into specific regions.

To conclude, despite public health and governmental efforts to increase parental adherence to recommendations, a high proportion of parents do not follow nutritional guidelines during the CF period. Altogether, these findings reinforce the need for public health interventions that are adapted to the current socio-cultural and economical context as a way of improving parental feeding practices during this crucial developmental period. Clinicians must also consider the multitude of factors affecting CF practices including parental education, financial resources, ethnicity, culture, and food environment when providing guidance to parents.

## Figures and Tables

**Table 1:** Summary of the timing of CF introduction and associated factors across continents.

Continent	Timing of CF * Introduction	Main Factors Associated with Very Early or Early CF Introduction
North America	In the United States, ~30% of infants were introduced to CF very early (<4 months) [10]; very early CF was less prevalent among families with a higher socio-economic status (4–7% vs. 20–48%) [52,53,54,55,56]. In one study in Canada, 28% of parents introduced CF very early [11]. In Hawaii, one study conducted among Native Hawaiian, Latino, and Pacific Islanders showed low prevalence of early CF (6%) [57].	Formula-feeding [10,53,58,59,60]. Lower socio-economic status [10]. Lower maternal education [60]. Maternal smoking [54]. Younger maternal age [60]. Higher maternal pre-pregnancy BMI ** [60]. Non-Hispanic Black race/ethnicity [10].
South America	In Chile [15,16] and Mexico [61], highly educated mothers tended to introduce CF ≥ 6 months. In Brazil [62] and Mexico [14], 29–48% of parents of lower socio-economic classes introduced CF < 4 months of age.	Shorter breastfeeding duration [12]. Lower socio-economic status [12]. Lower maternal education and prenatal advice [12,14]. Younger maternal age [12]. Smaller infant’s weight [14].
Europe	Overall, the prevalence of very early CF in Europe ranged between 0 and 34% [18,19,20,21,22,23,24,25,26,27,28,29,30,31]. Among six European countries, the median age of CF introduction was 6 months. Timing varied between countries: the highest prevalence of very early CF was in Belgium (18%), and delayed CF was more prevalent in Bulgaria (42%) [27]. Two studies conducted in the UK *** found a high prevalence of early CF among parents of White-British and Pakistani race/ethnicity [30,63]. The mean age for CF introduction ranged between 4.9 and 5.4 months in France [18,29,64,65] and 4.7 and 5.8 months in the UK [23,25,26,66,67,68,69].	Formula-feeding and/or shorter breastfeeding duration [17,21,22,26,29,31]. Lower socio-economic status [21,25,28]. Lower maternal education and knowledge on appropriate CF [17,21,22,27,29,31,70]. Younger maternal age [17,21,22,28,29,31,70]. Maternal smoking [17,21,27,29]. Higher infant weight [17,23,26]. Higher maternal weight [23,29]. Gestational age at birth (mixed findings) [17,22]. Parity (mixed findings) [17,31].
Oceania	Prevalence of very early CF introduction was low in Australia (1–24%) [32,71,72,73,74]; data from the 2020–2021 National Health Survey showed that 54% of children were introduced to CF ≥ 6 months [74] and two other studies found a median age of introduction of 5 months [32,73]. The mean age for CF introduction was 5.2–5.6 months among highly educated cohorts in Australia [71] and New Zealand [75].	Formula-feeding [73]. Lower maternal education [73]. Younger maternal age [73]. Maternal smoking [73]. Not married/partnered [73]. Mothers born in Australia (vs. born in Asia) [73]. Staying at home or student mothers (vs. working) [73].
Asia	The prevalence of very early CF was low (1–8%) in China [40], India [34], Lebanon [41], and the United Arab Emirates [42]. In China, 87% of parents from the Jiaxing Birth Cohort introduced CF > 6 months [39]. Introduction of CF at or after the recommended age was also observed in India [34,36], Thailand [37], and the United Arab Emirates [38,42]. Delayed CF was particularly prevalent among low-middle class Pakistani mothers (42%) [35], as well as in Bangladesh [76,77] and Nepal [78], where ~25% of infants were still not introduced to solids by 8 months.	Higher or lower maternal education (mixed findings) [35,39,40]. Farming as an occupation [39]. Living in a rural area (Pakistan) [35].
Africa	In Ethiopia, the prevalence of early CF was low (6–21%), and delayed CF was observed in 18–35% of infants [43,44,45]. In Nigeria [51], Zanzibar [79], and Cape Town [80], a higher proportion of parents introduced CF early (15–20% < 4 months in Cape Town) [80].	Residing in urban areas [80]. Lack of knowledge on appropriate CF timing [51].

* CF: Complementary feeding. We defined very early CF as CF < 4 months, early CF as CF < 6 months and delayed CF as CF > 6 months. ** BMI: Body-Mass Index. *** UK: United Kingdom.

**Table 2:** Summary of the timing of introduction of peanuts and eggs across continents.

Continent	Timing of Introduction of Peanuts and Eggs
North America	In the United States and in Canada, eggs and peanuts were generally not introduced at the beginning of CF *. Most parents introduced peanuts > 12 months of age [58,88]. In a Canadian qualitative study conducted among mothers of Middle Eastern origin, nuts were reported to have been introduced at a median age of 5.5 months [94].
South America	In Chile and in Mexico, eggs were not introduced at the beginning of the CF period, with ~50% of parents introducing them at 11–12 months [16,61].
Europe	In Poland, eggs and nuts were introduced between 8 and 12 months in a cohort of highly educated mothers [31]; another study showed that omnivore mothers (as opposed to vegetarian) introduced eggs earlier at the beginning of CF [70]. In Spain, the median age of egg introduction was 10 months, and they were only offered ≤ 2 times per week in most infants < 12 months [89]. In the UK ** [28] and in Latvia [95], 27–48% of infants aged 7 or 8–12 months did not consume eggs.
Oceania	In Australia, eggs and peanuts were introduced ~6–7 months [32].
Asia	In China, eggs were one of the first solid foods to be introduced [91,96] (at a mean age of 6 months in a nationally representative survey) [97]. Egg yolks were introduced before egg whites/whole eggs in China [91], Thailand [37], Taiwan [98], and Lebanon (although they were introduced much later in Lebanon) [41]. In Malaysia, one study found no egg consumption during a 2-day assessment in infants aged < 12 months [92]. In China [91] and Taiwan [98], only ~15% of children were introduced to peanuts in the first 12 months.
Africa	In Cape Town, eggs and peanuts were introduced at a median age of 12 months, however, 53% of infants aged 12–36 months living in rural areas were never exposed to peanuts (as compared to 6% in urban areas) [80]. In Tanzania and Senegal, ~5% of infants aged 6–11 months consumed eggs during a 24 h dietary recall [99]. Another study conducted in Ethiopia found a higher egg consumption among infants 6–11 months old (35%) [44]. The consumption of peanuts specifically was not assessed, however, 15% of infants aged < 24 months consumed “legumes and nuts” in Senegal [100] and 65% in Ethiopia based on 24 h dietary recalls [44].

* CF: Complementary feeding. ** UK: United Kingdom.

**Table 3:** Summary of the progression of food textures and prevalence of baby-led weaning across continents.

Continent	Progression of Food Textures and Prevalence of Baby-Led Weaning
North America	In the United States, 51–63% of infants were exposed to lumpy textures before the ages of 9–10 months [102,104]. In the United States, low-income mothers still offered purees > 12 months, especially mothers of Hispanic ethnicity (49% at 13 months) [103].
Europe	In France, ≥90% of parents offered only purees to infants aged < 8 months [18,64] and infants aged > 2 years still largely consumed pureed foods [64,65]. Only 2% reported using baby-led weaning [64]. Delayed introduction of multi-textured foods was associated with several parental and infant characteristics, such as older maternal age, later timing of CF *, higher apprehension towards introducing food textures and lower perception of infant’s readiness, higher offering of commercial baby foods (at 12–15 months), and eating only with the caregiver (as opposed to with the whole family) [64]. In Italy, the prevalence of baby-led weaning was 7–9% depending on the definition used. Baby-led weaning was associated with longer breastfeeding duration and timelier introduction of CF, and low puree-feeding was associated with higher infant birth weight and longer maternity leave [19,105]. In Poland and Austria, 13–19% of parents reported using baby-led weaning [106]. In Spain, pureed foods were also largely consumed by infants > 12 months, mainly in the form of pureed fruits [89]. In the UK **, 55% of 8–12 months infants were self-feeding finger foods [28].
Oceania	In New Zealand, one study conducted among a highly educated sample of participants showed that 18% of parents adhered fully to baby-led weaning and began using this approach at a mean age of 5.8 months [75].
Asia	In India, infants consumed foods from family meals at the beginning of CF [34]. In Nepal, 53% of infants aged 4–5 months had consumed a soft-solid food during a 24 h dietary recall [49]. In Thailand, most infants aged 6–8 months consumed pureed-mashed foods; self-feeding was not prevalent [37].

* CF: Complementary feeding. ** UK: United Kingdom.

**Table 4:** Summary of the consumption of iron-rich foods, cow’s milk, and tea at the beginning of the CF period and their associated factors across continents.

Continent	Consumption of Iron-Rich Foods, Cow’s Milk, and Tea	Associated Factors
North America	In Hawaii, the consumption of iron-rich foods was lower than in the United States; only 28% started CF * with iron-fortified cereals [57]. In the United States, the consumption of iron-rich foods was high among parents enrolled in the WIC federal program **: daily iron intake was ~13 mg at 7 months and 82% of parents started CF with iron-fortified cereals. Meat was introduced ~8 months and cow’s milk ~11 months [56,58,103]. In the United States, among a highly educated sample and with more Non-Hispanic White parents, only 25% of infants aged 6–8 months consumed meat and non-dairy proteins during a 24 h recall [59].	Earlier introduction of iron-fortified cereals: Non-Hispanic ethnicity or born in the United States [56]. Primiparity [56]. Mother unmarried [56]. Formula feeding [56,58]. Earlier introduction of meat: Non-Hispanic/non-Latino ethnicity or born in the United States [56]. Higher income [56]. Breastfeeding [58].
South America	In Brazil, meat was introduced lastly at ~8 months [13]. In a 24 h recall, half of infants aged 6–12 months consumed iron-rich foods [109]. In Mexico, 29% started CF with iron-fortified cereals and 26% with animal-based foods in a highly educated cohort [61]. Tea was introduced <12 months in 40–60% of infants in Brazil [12] and Mexico [61].	
Europe	In Europe, tea was commonly offered early during the CF period (at a median age of 3 months in an analysis of six European countries) [27]. In France, early consumption of cow’s milk was common [18]; in a study, 54% of parents reported giving cow’s milk to their 5–6 months old infants [110]. In Italy [20] and Poland [70], very few infants were on a vegetarian diet (even those of mothers adhering to vegetarianism). In Spain [89] and in France [18], iron-fortified cereals were introduced early and meat was introduced at ~7 months of age. In the UK ***, the consumption of iron-rich foods, tea and cow’s milk seemed low in the first 12 months of age [25,28,66,67,69].	Higher consumption of iron-fortified cereals: Traditional spoon-feeding method [66,67,69]. Later introduction of cow’s milk: Higher maternal education [31]. Higher knowledge of appropriate CF practices [31]. Primiparity [31]. Food allergies/family atopy history [31]. Maternal vegetarian diet [70]. Higher meat consumption: Shorter breastfeeding duration [20]. Maternal omnivore diet [70]. Living in a less deprived area [25].
Oceania	In New Zealand, 48% of infants (highly educated parents) consumed iron-fortified cereals at 6 months [75] and in Australia, 75% of parents began CF with cereals (not specified whether fortified or not) [111]. In Australia, daily iron intake was ~7 mg at 12–14 months, and meat only contributed to 6% of this intake [112].	Higher iron intake: Formula feeding [112]. Primiparity [112]. Higher consumption of iron-fortified cereals: Traditional spoon feeding [75]. Higher consumption of meat: Baby-led weaning [75].
Asia	In Bangladesh, 67% of infants consumed animal proteins during CF [76]. In Nepal, 15% of infants aged 6–11 months consumed tea/coffee and 32% iron-fortified cereals. Additionally, the consumption of meat and seafood was low in Nepal but high in Cambodia (74%) [99]. In China, 78% of parents introduced iron-fortified cereals ≤ 6 months [40]. In a nationally representative survey, meat was introduced ~9 months [97] (similarly to Lebanon [41]). In Lebanon, 54% of infants consumed iron-fortified cereals at the beginning of CF and 57% of infants were introduced to tea < 4 months old [41]. In India, 22% of infants consumed iron-rich animal products at 12 months [93]. Daily iron intake at 6–11 months was 10.3 mg in the United Arab Emirates (47% had intakes < 6.9 mg/day) [42] and 6.2 mg **** in Malaysia [92]. In Sri Lanka, nearly none of the parents began CF with iron-fortified cereals and 31% had offered “plain” tea < 12 months [50]. In Thailand, ~13% of parents (highly educated cohort) introduced cow’s milk < 12 months [37].	
Africa	In Senegal, 49% of infants 6–11 months old consumed iron-rich foods [100]. In Zanzibar, Tanzania, daily iron intake from foods averaged 1.74 mg [79]. Plain and sweetened tea were commonly offered during CF in Tanzania [79,99].	Higher consumption of iron-rich foods: Lower household food insecurity [100].

* CF: Complementary feeding. ** WIC: “The Special Supplemental Nutrition Program for Women, Infants, and Children” in the United States [56]. *** UK: United Kingdom. **** This study excluded infant formulas which can be a significant contributor to daily iron intake at this age.

**Table 5:** Summary of the consumption of added sugars, juice, and honey during the CF period and its associated factors across continents.

Continent	Consumption of Added Sugars during the CF * Period	Associated Factors
North America	In Canada, Middle Eastern mothers commonly offered sweetened water during their child’s first months of life [94]. In the United States, added sugars were primarily consumed through ultra-processed foods (e.g., sweetened yogurt, commercial infant snacks, pastries, juice) [56,116]. In a nationally representative study, 61% of infants aged 6–11 months already consumed added sugars [116] while other studies showed that 60–93% of infants < 12 months consumed added sugars [52,55,56]. The consumption of added sugars < 12 months decreased over recent years among women enrolled in the WIC ** federal program [56]. In low-income cohorts in the United States, 20–50% of infants consumed fruit juice during the first 6 months of life [54,56].	Non-Hispanic Black and non-Latino race/ethnicity [56,59,116]. Born in the United States [56]. Mother unmarried [56]. Younger maternal age [56].
South America	Many studies have assessed the consumption of added sugars in Brazil, which are consumed directly (added to meals) and through ultra-processed foods (breakfast cereals, sweetened dairy products, juice, cookies, etc.) regularly <12 months. The prevalence of ultra-processed foods introduction during the CF period (<24 months) in Brazil was 79–96% of infants [13,117,118]. In Brazil, although the early consumption of juice seems to be decreasing over the years, it remains problematic: 13–43% of infants had been exposed to juice < 6 months [12,13] and the median age of juice introduction was 4 months in another study [47]. Similar findings were also obtained in Mexico [14]. In Chile, 9% of parents added sweeteners to foods < 24 months (including honey), 56% offered cookies and 20% offered juice frequently [15]. In Haiti, ~¼ of infants consumed sweetened traditional drinks in the first days of life [119].	Formula feeding and/or shorter breastfeeding duration [109,118,120]. Lower income [62,117,121]. Number of people in the household (mixed findings) [117,120]. Lower maternal education [62,121,122]. Multiparity [121]. Older maternal age [121].
Europe	In Europe, juice was introduced at a median age of 6 months [27]. In the UK ***, a national survey showed that 29% of parents had offered ultra-processed foods including chocolate and ice cream to 8–12 months old infants [28]. The addition of sweeteners to complementary foods was prevalent in Europe; in France, 9% of parents added sugars to complementary foods at the age of 6 months [123]. This increased to one-third of infants at 12–24 months in Poland and Austria [106]. In the UK, Pakistani and Bangladeshi parents commonly offered sweet foods and beverages, as well as traditional sweetened meals (i.e., ghutti containing honey), during CF [30,63].	Pakistani/Bangladeshi and Other South Asian race/ethnicity [30]. Infant feeding method (mixed findings) [106,123]. Very early CF introduction [123]. Multiparity [106]. Younger maternal age [28,106]. Living in rural and/or more deprived areas (mixed findings) [25,28,106]. Lower income [106]. Traditional spoon-feeding [66]. Relying on personal experience/family for information [123].
Oceania	One study conducted in Australia showed that 96% of infants aged ≤ 24 months consumed ultra-processed foods [72].	Younger maternal age [72]. Primiparity [72]. Mothers born in Australia and the UK (vs. born in Asia) [72].
Asia	The consumption of ultra-processed foods was also high in Asia, where almost all infants were introduced to sweet/salty commercial snacks by 24 months of age in China [33], India [93], Indonesia [124], Lebanon [41] and Nepal [49,99]. The consumption of juice and other sweetened beverages during the CF period was lower than other ultra-processed foods but remained high in the United Arab Emirates (52%) [38] and in Thailand where 26% of infants aged 6–8 months consumed 1–2 oz of fruit juice/day [37]. Parents frequently added sugar to complementary foods, notably in Nepal (52% of children aged < 24 months) [49] and Sri Lanka where salt and sugar were introduced at ~7.6 months [50]. In Nepal and Cambodia 23–35% of infants aged 6–11 months consumed sugar or honey in their foods/drinks [99]. In one study in India, 9% of mothers gave pre-lacteal feeds (most often sweetened) to their infants [93].	Lower income [99]. Lower maternal education [124]. Higher parental emotional/instrumental behaviors during CF **** [33]. Not offering formula [124]. Not offering commercial infant foods [124].
Africa	Consumption of added sugar through foods or drinks was frequent in Africa; 50–70% of parents of infants aged 6–11 months from Senegal and Tanzania added sugar or honey to complementary foods [99]. In Zanzibar, Tanzania, most infants consumed sweetened tea < 24 months [79].	Lower maternal education [99]. Not offering formula [99].

* CF: Complementary feeding. ** WIC: “The Special Supplemental Nutrition Program for Women, Infants, and Children” in the United States [56]. *** UK: United Kingdom. **** “Emotional feeding indicates using foods to calm or soothe, or for boredom or fussiness, and instrumental feeding indicates using foods to reward or punish” [33].

## Data Availability

No new data were created or analyzed in this study. Data sharing is not applicable to this article.

## Supplementary Materials

The following supporting information can be downloaded at: https://www.mdpi.com/article/10.3390/children10050794/s1, Table S1: Detailed timing of CF introduction and its associated factors across continents; Table S2: Detailed age at introduction of peanuts and eggs and its associated factors across continents; Table S3: Detailed progression of food textures, prevalence of baby-led weaning and their associated factors across continents; Table S4: Detailed consumption or iron-rich foods, cow’s milk and tea and their associated factors across continents; Table S5: Detailed consumption of added sugars and its associated factors across continents.
