# Supplementary material for: Are Complementary Feeding Practices Aligned with Current Recommendations? A Narrative Review
Source: Children (Basel). 2023 Apr 28;10(5):794. doi: 10.3390/children10050794 (PMC10217166; doi:10.3390/children10050794)
Supplement: Supplementary file 1 [file children-10-00794-s001.zip › children-2335472-supplementary.pdf]

Supplementary files

Table S1. Detailed timing of CF[1] introduction and its associated factors across continents

Con-tinent	Setting (country, n, sample characteristics)	Timing of CF introduction	Factors Associated with Very Early or Early CF introduction
North America	Canada, n = 22, Middle Eastern mothers, low socio-economic status [1]	< 4 months: 7/22 [2]<br>< 6 months: 19/22	- Fear of judgement when BF [3] in public<br>- Price of FF
	Canada, n = 392, representative of mothers from Southeastern Quebec [2]	< 4 months: 28%	-
	Hawaii, n = 70, Native Hawaiian, Filipino and Pacific Islander parents [3]	< 4 months: 6%<br>< 6 months: 53%	-
	United States, n = 115, mostly middle-class, Caucasian, married mothers with multiple children [4]	< 16 weeks: 26%<br>Mean: 4 months	- Younger maternal age<br>- Higher maternal pre-pregnancy BMI<br>- Lower maternal education<br>- FF at 4 months
	United States, n = 3777, nationally representative sample of mothers enrolled in the WIC federal program [4] (low-income) [5]	< 4 months: 20%	-
	United States, n = 3235, slightly higher proportion of White non-Hispanic vs Hispanic parents and education than US population [6]	< 4 months: 17% [5]	- FF
	United States, n = 449, mostly low-income mothers of non-Hispanic Black race/ethnicity [7]	< 4 months: 32%	-
	United States, n = 23 927, nationally representative sample [8]	< 4 months: 32%<br>4-6 months: 51%<br>Mean: 4.7 months	- Non-Hispanic Black race/ethnicity<br>- EFF at 4 months<br>- Lower socio-economic status
	United States, n = 2839, nationally representative sample of mothers enrolled in the WIC federal program [9]	Mean: 4.6-5.2 months	- FF
	United States, n = 443, low income and education, mostly of non-Hispanic White and Black race/ethnicity [10]	< 4 months: 48%<br>< 6 months: 83%	- Maternal smoking<br>- Working from home
	United States, n = 141, highly educated families of Caucasian/race ethnicity [11]	< 4 months: 4%<br>< 6 months: 48%	- FF
	United States, n = 217, mostly mothers of non-Hispanic White race/ethnicity with high education levels and income [12]	< 4 months: 7%<br>Mean: 4.9 months	- Lower infant "enjoyment" of BF
	United States, n = 328, mothers from the Grand Rapids metropolitan area [13]	Mean: 5.4 months	-
South America	Brazil, n = 1567, low education, 1/3 having ≤ minimum wage [14]	< 4 months: 48% (inappropriate CF) [6]	-
	Brazil, n = 79, low income and education [15]	< 6 months: 40/79<br>6 months: 29/79<br>Mean: 5.3 months	-

	Brazil, n = 3823 (2004); n = 3689 (2015), mostly low-middle education [7], equally distributed wealth levels [16]	< 6 months: 93% (2004); 87% (2015)	- Shorter BF duration<br>- Younger maternal age<br>- Lower maternal education<br>- Lower income
	Chile, n = 261, highly educated mothers [17]	Median: 6.4 months	-
	Chile, n = 85, highly educated Chilean mothers, mostly > 30 years old [18]	6 months: 85%	-
	Mexico, n = 377, highly educated [19]	Mean: 6.1 months	-
	Mexico, n = 143, Mexican mothers with low socio-economic status and beneficiaries of governmental social security [20]	< 4 months: 29%<br>< 6 months: 78%	- Younger maternal age<br>- Lack of prenatal advice on the timing of CF and lower infant size
	Belgium, Bulgaria, Germany, Greece, Poland and Spain, n = 6800 [21]	< 4 months: 1-18%<br>4-6 months: 56-84%<br>> 6 months: 8-42%<br>Median: 6 months	- Low socioeconomic status/ maternal education [8]<br>- Smoking during pregnancy (CF < 4 or > 6 months)
	Denmark, n = 4503 [22]	< 4 months: 7%<br>4-6 months: 65%<br>> 6 months: 28%	- Primiparity<br>- Not fully BF at 5 weeks<br>- Lower maternal education<br>- Maternal smoking<br>- Higher gestational age at birth and infant birth weight<br>- Younger maternal age
	England, n = 1327, multi-ethnic cohort (White British, Pakistani, Other South Asian, and Other race/ethnicities) [23]	< 4 months: 27%	- White British race/ethnicity (as compared to Pakistani and Other South Asian)
	France, n = 10931, 72% ≥ 12 years of education, predominantly born in France [24]	< 4 months: 26%<br>4-6 months: 62%<br>> 6 months: 12%<br>Mean: 5.2 months	- Maternal smoking<br>- Higher maternal weight<br>- Younger maternal age<br>- Lower maternal education<br>- Born outside of France<br>- Shorter BF duration<br>- Not attending prenatal information classes<br>- Longer BF duration and 2nd infant (associated with CF > 6 months)
	France, n = 1184, comparable with the population of France (mainland)[25]	< 4 months: 6%<br>4-6 months: 85%<br>> 7 months: 10%<br>Mean: 5.4 months	-
	France, n = 2999, highly educated mothers born in France [26]	Mean: 4.9 months	-
	France, n = 181, highly educated [27]	Mean: 5.3 months	-
	Italy, n = 360, ~ half of mothers with higher education [28]	< 4 months: 3%<br>4-6 months: 49%<br>> 6 months: 49%	-
**Europe**	Italy, n = 1245, high level of maternal education, mostly married/partnered [29]	≥ 6 months: 65.1%<br>Mean: 5.7 months	-

	Italy, n = 2023 (Northern Italy: n = 584; Central: 1230; Southern: 209) [30]	< 5 months: 13%<br>5-6 months: 77%<br>> 6 months: 10%	-
	Norway, n = 715, highly educated mothers, slightly more than half of them primiparous [31]	< 4 months: 5%<br>4-5.5 months: 81%	- EFF at 3 months<br>- Younger maternal age<br>- Not married/cohabiting<br>- Maternal smoking<br>- Lower maternal education<br>- Economic difficulties<br>- Not EBF ≤ 1st month
	Poland n = 4065 and Austria n = 1750, highly educated mothers [32]	< 4 months: 2-4%<br>4-6 months: 61-75%<br>> 6 months: 20-37%	- Younger maternal age<br>- Lower maternal education<br>- Shorter pregnancy duration<br>- FF
	Poland, n = 289, mothers predominantly having a university degree, most with 1-2 children and "medium" knowledge about appropriate CF practices [33]	< 4 months: 34%<br>4-6 months: 63%<br>> 6 months: 4%	- EFF, multiparity, lower maternal education and CF knowledge, maternal age < 35 years<br>- EBF, higher CF knowledge, maternal age > 35 years (associated with delayed CF)
	Poland, n= 251, ~ 3/4 with a university degree and ~ half of them vegetarian [34]	Mean: 3.7-4.2 months	- Low knowledge on CF<br>- Adhering to a traditional (omnivore) diet<br>- Younger maternal age (< 25 years)
	United Kingdom, n = 604, mothers with ~ 14 years of education, mostly primiparous [35]	< 4 months: 22%<br>< 6 months: 66%<br>Mean: 4.8 months	- Higher maternal anxiety and symptoms of obsessive-compulsive disorder<br>- Higher restrained and emotional eating behaviors<br>- Higher maternal BMI<br>- Higher infant weight
	United Kingdom, n = 2747, national survey conducted in Scotland [36]	< 4 months: 3%<br>4-6 months: 51%<br>≥ 6 months: 46%	- Younger maternal age<br>- Living in a deprived area
	United Kingdom, n = 134, predominantly mothers of White race/ethnicity with > 80% having a university degree [37]	Mean: 5.5-5.8 months	-
	United Kingdom, n = 64, mostly highly educated although ~ half living in deprived areas [38]	< 4 months: 3%<br>6 months: 34%<br>Mean: 5 months	- Residing a more socio-economically deprived area
	United Kingdom, n = 565, more than half occupying managerial positions [39]	Mean: 5.7 months	-
	United Kingdom, n = 72, mostly White British race/ethnicity and ~ half having a university degree [40]	< 4 months: 11% (n = 8)<br>≥ 6 months: 8% (n = 6)<br>Mean: 4.7 months	- Higher infant birth weight<br>- Shorter BF duration<br>- Happier infant temperament<br>- Less maternal "verbal involvement" during meals<br>- Less restricting behaviors during BF

	United Kingdom, n = 110, parents of Bangladeshi, Pakistani, White British, Polish, Black African and Caribbean race/ethnicity residing a deprived area [41]	Pakistani, Bangladeshi, and Polish: 3-5 months<br>White British: 4-5 months<br>Black African and Caribbean: 6 months	-
	United Kingdom, n = 60, household income primarily ~ 40-60K, ~ 43% with an undergraduate degree [42]	< 4 months: 0-3%<br>4-6 months: 30%<br>6 months: 67-70%	-
	United Kingdom, n = 131, majority of White British race/ethnicity [43]	Mean: 5.6 months	-
	United Kingdom, n = 96, highly educated, mostly occupying managerial work positions [44]	Mean: ~ 5.5 months	-
**Oceania**	Australia, n = 206, highly educated, predominantly partnered, and most not working full time[45]	< 4 months: 5%<br>4-6 months: 47%<br>≥ 6 months: 48%<br>Mean: 5.6 months	-
	Australia, n = 828, comparable to the maternal population of South Australia; mostly born in Australia/New Zealand and more than half highly educated [46]	< 4 months: 24%<br>4-5 months: 66%<br>≥ 6 months: 9%	-
	Australia, n = 934 [47]	< 4 months: 14%<br>< 6 months: 77%<br>Median: ~ 5 months	- Younger maternal age<br>- Lower maternal education<br>- Non-married/partnered<br>- Maternal smoking<br>- Maternal country of birth (born in Australia)<br>- Maternal occupation (stay-at-home/students)<br>- FF at 4 weeks + shorter BF
	Australia, n = 4981, high maternal education level [48]	4 months: 21%<br>5 months: 24%<br>6 months: 46%	-
	Australia, n = ~11 000 children aged 0-3 years included in the 2020-21 National Health Survey [49]	< 4 months: 5%<br>4-5 months: 41%<br>≥ 6 months: 54%	-
	Australia, n = 1140, comparable to the Australian population although more highly educated and with a higher economic status [50]	Median: 5 months<br>< 4 months: 1 %<br>> 6 months: 3%	-
	New Zealand, n = 876, highly educated [51]	Mean: 5.2 months [9]	-
**Asia**	Bangladesh, n = 400, mostly young mothers with low education levels, predominantly identifying as housewives and Islamic. Mild to moderate food insecurity was prevalent among the families [52]	76% of infants aged 6-8 months received CF	-
	Bangladesh, n = 2167 (2017-2018), nationally representative [53]	75% of infants aged 6-8 months received CF	-
	China, n = 18446, Jiaxing Birth Cohort [54]	< 6 months: 13%<br>≥ 6 months: 87%	- Lower maternal education<br>- Farming (occupation)

	China, n = 21, mostly highly educated, primiparous mothers [55]	4 months: 5%<br>5 months: 14%<br>6 months: 71%<br>8 months: 10%	-
	China, n = 760, mostly highly educated employed mothers (around 50% intended to come back to work ≤ 6 months post-partum) [56]	< 4 months: 7%<br>< 6 months: 94%<br>Median: 5 months	- Higher maternal education
	China, n = 408, mostly ≤ senior middle school education level, around half of them unemployed [57]	< 6 months: 15%<br>6-8 months: 81%<br>> 8 months: 3%	-
	China, n = 2251, predominantly mothers of Han Chinese ethnicity, > half with ≥ 16 years of education [58]	Mean: 5.5 months<br>5-6 months: > 80%	-
	India, n = 297, married mothers from the Santal tribe with low education levels [59]	< 4 months: 1%<br>6-8 months: 82%<br>9-12 months: 10%<br>Mean: 6.8 months	-
	India, n = 902, mostly stay-at-home mothers with secondary education [10] [60]	Median: 5.5 months<br>6 months: 36%	-
	Lebanon, n = 1051, representative of Lebanese children enrolled in daycare centers, mostly highly educated married parents [61]	< 4 months: 8%<br>4-5 months: 45%<br>≥ 6 months: 46% [11]	-
	Nepal, n = unspecified, nationally representative [62]	In 2016, 77% of infants aged 6-8 months received CF	-
	Pakistan, n = 202, mostly low-middle class stay-at-home mothers with low education living in an urban area [63]	< 6 months: 10%<br>6 months: 44%<br>> 6 months: 42%	- Lower education level<br>- Living in a rural area<br>(for CF < or > 6 months)
	South India, n = 95, highly educated, mostly middle-class mothers identifying as housewives [64]	6 months: 91%	-
	Sri Lanka, n = 515, mostly residing in rural areas, identifying as housewives [65]	4 months: 12%<br>6 months: 49%	-
	Taiwan, n = 272 [66]	4-6 months: 85%<br>Mean: 5.4 months<br>Median: 6 months	-
	Thailand, n = 108, mostly middle-class, highly educated participants (mother or family member) [67]	< 6 months: 11%<br>6 months: 60%<br>> 6 months: 28%	-
	United Arab Emirates, n = 1822, predominantly married, highly educated mothers [68]	< 6 months: 28%<br>≥ 6 months: 72%	-
	United Arab Emirates, n = 276, more than half of parents highly educated [69]	< 4 months: 7%<br>< 6 months: 12%<br>6-8 months: 79%	-
**Africa**	Cape Town, n = 1185 (urban) + 398 (rural) [70]	< 4 months: 15-20%<br>4-6 months: 55-69%<br>> 6 months: 11-30%<br>Median: 6 months	- Residing in an urban area

Ethiopia, n = 3433, very low education, mostly married, identifying as housewives and living in a rural area [71]	< 6 months: 6%<br>6 months: 60%<br>> 6 months: 35%	-
Ethiopia, n = 323, mothers of Oromo ethnicity, mostly married, living in rural area and with low education [72]	< 6 months: 21%<br>6 months: 60%<br>> 6 months: 18%	-
Ethiopia, n = 520, mostly multiparous, Islamic, married mothers with low education, 22% with food insecurity [73]	< 6 months: 6%<br>6 months: 68%<br>> 6 months: 26%	-
Nigeria, n = 135, mostly married Christian mothers of Yoruba ethnicity with education ≥ secondary level [74]	1 month: 3%<br>3-5 months: 63%<br>≥ 6 months: 31% [12]	- Lack of knowledge on appropriate timing of CF
Zanzibar, Tanzania, n = 200 [75]	Mean: 4 months	-

[1] CF: Complementary feeding

[2] Represents the introduction of table foods (excluding drinks). Moreover, 3 / 22 mothers with children < 6 months had not yet introduced table foods.

[3] BF: Breastfeeding, EBF: Exclusive Breastfeeding, FF: Formula-feeding, EFF: Exclusive Formula-feeding

[4] WIC: Special Supplemental Nutrition Program for Women, Infants, and Children.

[5] On the day of the recall.

[6] Introduction of "inappropriate" CF based on the Brazilian food guide and including chocolate flavored milk, candies, snacks and other ultra-processed foods.

[7] The proportion of mothers with ≥ 12 years of education increased in the 2015 cohort as compared to 2004 (31 vs 10%).

[8] In this study, low socioeconomic status was defined as low maternal level of education.

[9] 26 children were not introduced to CF by the age of 6-7 months and were excluded from the study.

[10] Participants had already undergone a RCT during pregnancy and postpartum which assessed the effect of DHA supplementation on child neurodevelopment.

[11] Data only represents the introduction of solid foods (excluding liquids).

[12] Age at introduction of solid foods including "formulated foods" but excluding water. More than 3/4 of infants were introduced to water before the age of 6 months.

Table S2. Detailed age at introduction of different food allergens across continents

Continent	Setting (country, n, sample characteristics)	Timing of Introduction of Peanuts and Eggs	Associated Factors
North America	Canada, n = 22, Middle Eastern mothers with a low socio-economic status [1]	Peanuts and others nuts: median of ~ 25 weeks	-
	Canada, n = 2124, predominantly Caucasian parents with a high socio-economic status [76]	Introduction of<br>Eggs > 12 months: 21%<br>Peanuts > 12 months: 63%	-
	United States, n = 2839, nationally representative sample of mothers enrolled in the WIC federal program [1] [9]	Eggs: 9.6-10.5 months<br>Peanut butter: 12.2-13 months	- EFF[2] among girls (associated with later introduction of peanut butter)
South America	Chile, n = 85, highly educated Chilean mothers [18]	Eggs: 46% introduced eggs at 11 months	-
	Mexico, n = 377, highly educated mothers [19]	Introduction of eggs: ~ half ≥ 12 months	-
Europe	Italy, n = 360, More than half of mothers with higher education [28]	2% of infants consumed a vegan diet (no eggs) during the CF [3] period	-
	Latvia, n = 266, mothers with secondary-university degree [77]	Consumption of eggs: 5% of infants aged 4-6 months and 61% of 7-12 months infants (median of 1 egg/week)	-
	Poland, n = 289, predominantly mothers with a university degree, most with 1 or 2 children and "medium" knowledge in appropriate CF practices [33]	Mean age at introduction of eggs, fish and nuts: 8-12 months	- Lower maternal education and nutrition knowledge (associated with introduction of nuts > 6 months)
	Poland, n= 251, predominantly mothers with a university degree and a high proportion adhering to vegetarianism (44%) [34]	Eggs: > 50% around 8-9 months in vegetarian mothers. In omnivore mothers, eggs were introduced ~ 4.6 months	-
	Spain, n = 630, predominantly married/partnered mothers with a university degree, 60% working full time [78]	Median age of introduction:<br>Eggs: 10 months (offered ≤ 2 times per week in 87% of infants aged 7-11 months)	-
	United Kingdom, n = 2747, national survey conducted in Scotland [36]	27% of infants aged 8-12 months never consumed eggs, 42% ≤ once/week and 23% 2-4 times/ week	-
Oceania	Australia, n = 1140, comparable to the Australian population although more highly educated and with a higher income [50]	Median age of introduction:<br>Eggs: 6 months<br>Peanuts: 7 months<br>87% introduced eggs ≤ 9 months and 83% for peanuts	-
Asia	China, n = 21, mostly highly educated, primiparous mothers [55]	Eggs were among the first common foods during CF	-

	China, n = 89006 (2015), nationally representative [79]	Mean age of introduction (for ≥50% of children) for eggs: 6 months	-
	China, n = 760, mostly highly educated employed mothers (~ half of them intending to go back to work ≤ 6 months post-partum) [56]	53% introduced eggs (and/or meat) ≤ 6 months	-
	China, n = 2251, predominantly mothers of Han Chinese ethnicity, more than half of them with ≥ 16 years of education [58]	Egg yolks: 40% ≤ 6 months<br>Eggs: 91% ≤ 12 months<br>Peanuts: 14% ≤ 12 months	-
	Lebanon, n = 1051, representative of Lebanese children enrolled in daycare centers, mostly highly educated married parents [61]	% of infants not introduced to whole eggs < 12 months: 72% (60% for egg yolks)	-
	Malaysia, n = 119, Malay mothers residing in an urban area [80]	Eggs: 0% of 6-11 months infants consumed eggs, and 39% of 12-17 months infants during a 2-day assessment	-
	Taiwan, n = 272 [66]	% of infants **not** introduced to peanut/eggs ≤ 12 months:<br>Peanuts: 85%<br>Egg yolk: 21% / whites: 37%	-
	Thailand, n = 108, mostly middle-class, highly educated mother or family member [67]	Egg yolk: first animal protein to be introduced	-
**Africa**	Ethiopia, n = 323, mothers of Oromo ethnicity, mostly married, living in rural areas and with low education levels [72]	Among children aged 6-11 months, 35% consumed eggs and 54% legumes/nuts during a 24h recall	-
	Cambodia (n = 222), Nepal (n = 228), Senegal (n = 218) and Tanzania (n = 229) predominantly married mothers with varying socio-economic status [81]	In a 24h recall, egg consumption was low among infants aged < 12 months, especially in Tanzania and Senegal	-
	Cape Town, n = 1185 (urban) + 398 (rural) [70]	Median age at introduction of eggs and peanuts: 12 months. 53% of rural infants were never exposed to peanuts (6% in urban infants)	- Residing in a rural area (lower exposure to allergenic foods)
	Senegal, n = 98, low education, high prevalence of food insecurity [82]	Low consumption of eggs (9%) and legumes/nuts (15%) in infants < 24 months (24h recall)	-

[1] WIC: Special Supplemental Nutrition Program for Women, Infants, and Children
[2] EFF: Exclusive Formula-Feeding
[3] CF: Complementary Feeding

Table S3. Detailed progression of food texture during the CF [1] period across continents

Continent	Setting (country, n, sample characteristics)	Progression of Textures	Associated Factors
North America	United States, n = 1261, nationally representative of mothers enrolled in the WIC federal program [2] [83]	51% of infants were introduced to lumpy foods by 9 months.	-
	United States, n = 3777, nationally representative of mothers enrolled in the WIC federal program [84]	Consumption of pureed foods at 9 months: 52%<br>11 months: 47%<br>13 months: 36%	- African American parents offer less purees but pre-masticate more; parents of Hispanic ethnicity offer more purees
	United States, n = 328 [13]	63% introduced lumpy textures < 9 months.	-
Europe	France, n = 1184, comparable to the mainland France population [25]	~ 90% of infants aged < 8 months consumed purees and ~ 50% of 8-11 months infants were introduced to lumpy textures	-
	France, n = 2999, highly educated mothers, predominantly born in France [26]	Only purees were offered to infants aged 4-7 months; cooked and soft pieces of foods were offered ≥ 8 months and large cooked/hard pieces, double textures from 10 months. Infants aged 2-3 years still consumed purees. Only 10% knew about BLW [3] and 2% reported using this approach	*Factors associated with lower exposure to multi-textured foods:*<br>- Older maternal age<br>- Later CF introduction<br>- Higher consumption of commercial baby foods (at 12-15 months)<br>- Eating with a caregiver (as opposed to with the whole family)<br>- Lower perception of infant's readiness and higher apprehension towards the introduction of food textures
	France, n = 181, predominantly highly educated mothers [27]	Age at introduction of semi-liquid purees: 5.5 months, mashed: 9.1 months, small soft pieces: 11.4 months and hard pieces of food: 14.4 months. Infants aged ≥ 24 months still largely consumed semi-liquid purees (70%)	-
	Italy, n = 1245, highly educated, mostly married/partnered [29]	29% of infants aged 6-12 months were fed purees ≥ 90% of the time and 33% ≤ 10% of the time. 40% were fed family foods ≥ 90% of the time, and 18% ≤ 10% of the time. 7% followed all 3 components of BLW.	- *Low puree-feeding:* high infant birth weight, longer maternity leave<br>- *High family foods*: longer maternity leave, lower maternal income

			- *BLW*: longer BF duration, timely CF introduction
	Italy, n = 2023 (Northern Italy: 584; Central Italy: 1230; Southern Italy: 209) [30]	77% of families used the TSF [4] method and 9% BLW	- BLW more prevalent in Northern Italy than in the other regions
	Poland, n = 4110 and Austria, n = 1783, highly educated (more so in Poland than in Austria) [85]	Most mothers used a mixed approach to CF (spoon + self-feeding). 13-19% used BLW.	-
	Spain, n = 630, predominantly married/partnered mothers with a university degree, most working full time [78]	66% of infants aged 12-18 months still consumed pureed fruits ≥ 5 days/week and 37% for pureed vegetables	-
	United Kingdom, n = 2747, national survey conducted in Scotland [36]	55% of children aged 8-12 months were self-feeding finger foods and 72% were fed "mashed, lumpy or pureed foods" > once/day	-
**Oceania**	New Zealand, n = 876, highly educated [51]	Partial BLW: 11%<br>Full BLW: 18%<br>Parents who tried BLW did so ~ 6.4 months (5.8 months for full BLW)	-
**Asia**	India, n = 297, married mothers from the Santal tribe with low education [59]	Family foods were offered at the beginning of CF	-
	Nepal, n = 309, predominantly married [86]	25% of infants aged < 6 months had consumed a soft to solid food the day of the recall (53% between 4-5 months)	-
	Thailand, n = 108, mostly middle-class, highly educated (mother or family member) [67]	Infants aged 6-8 months consumed mostly pureed or mashed foods and self-feeding was not prevalent	-

[1] CF: Complementary Feeding
[2] WIC: Special Supplemental Nutrition Program for Women, Infants, and Children.
[3] BLW: Baby-led Weaning
[4] TSF: Traditional spoon-feeding

Table S4. Detailed consumption of iron-rich foods, cow's milk and tea during the CF [1] period and their associated factors across continents

Continent	Setting (country, n, sample characteristics)	Consumption of Iron-Rich Foods, Cow's Milk and Tea	Associated Factors
North America	Hawaii, n = 70, Native Hawaiian, Filipino and Pacific Islander [3]	28% started CF with iron-fortified cereals (other 29%: traditional tuber-based dish)	-
	United States, n = 3777, nationally representative sample of mothers enrolled in the WIC federal program [2] (low-income) [5,84]	82% started CF with iron-fortified cereals (median age of introduction: 4.5 months)<br>Cow's milk: 37% ≤ 11 months (median age: 11.7 months)<br>Median iron intake at 7 months: 12.9mg/day [3] (20% had inadequate iron intakes)	*Earlier introduction of:*<br>- *Iron-fortified cereals*: Non-Hispanic ethnicity, US born, unmarried mothers, primiparity breast + formula-feeding<br>- *Meats:* Non-Hispanic/ Non-Latino ethnicity, US born, higher income
	United States, n = 1261, nationally representative sample of mothers enrolled in the WIC federal program [83]	15% of infants were exposed to tea by 7 months	-
	United States, n = 3235, slightly higher education levels and proportion of Non-Hispanic white parents than US population [6]	~ 50% of infants aged 4-12 were fed iron-fortified cereals and only 25% of those in the 6-8 months group consumed meats & other sources of proteins (excluding dairy) in a 24h recall	-
	United States, n = 2839, nationally representative sample of mothers enrolled in the WIC federal program [9]	Mean age at introduction of:<br>Iron-fortified cereals: 5-6.7 months,<br>meat: 7.6-8.5 months,<br>cow's milk: 10.6-11.5 months	*Earlier introduction of*:<br>- *Iron-fortified cereals*: EFF<br>- *Meat*: EBF [4]
South America	Brazil, n = 700 [87]	Among children aged ≤ 7.7 months, 10 % had been introduced to tea and/or cow's milk	
	Brazil, n = 79, low income and education [15]	Meat was usually introduced last during CF at ~ 8 months	-
	Brazil, n = 3823 (2004); n = 3689 (2015), mostly low-middle education [5], equally distributed wealth levels [16]	Tea was introduced < 12 months in 74% of children in 2004 and 60% in 2015.	-
	Brazil, n = 847, mostly mothers with low education levels, of black or brown race/ethnicity [88]	49% of infants (6-12 months) consumed iron-rich foods (undefined; based on 24h recall)	-
	Chile, n = 261, ~ half with > 16 years of education [17]	Among children aged ≤ 24 months, only 3% had been introduced to tea/coffee	
	Mexico, n = 377, highly educated mothers [19]	29% started CF with cereals (unspecified if fortified) and 26% animal-based foods, ~ 40% had introduced tea < 12 months	-

**Europe**	Europe, Belgium, Bulgaria, Germany, Greece, Poland and Spain, n = 6800 [21]	Tea was commonly offered at the beginning of CF (median age: 3 months)	-
	France, n = 1184, comparable with the population of France (mainland) [25]	Mean age at introduction of: Iron-fortified cereals: 5.6 months, meat/fish: 7.3 months. Consumption of cow's milk < 12 months: ~ 11-55g/day	-
	France, n = 600 [89]	At inclusion, 54% of infants aged 5-6 months consumed cow's milk (24h recall)	-
	Italy, n = 360, > half of mothers with higher education [28]	9% of infants did not consume meat and/or animal-based products during CF	- *Non-omnivore diet*: Longer BF duration
	Latvia, n = 266, mothers with secondary-university degree [77]	Meats were consumed by 10% of infants aged 4-6 months and 85% of 7-12 months' infants. Between 6-12 months, the median quantity was 46g x 1.7 times/day)	-
	Poland, n = 289, predominantly mothers with a university degree, most having 1 or 2 children and "medium" knowledge in appropriate CF practices [33]	Vegetables and fruit purees were the first CF introduced (not iron-rich foods). According to the authors, cow's milk was introduced earlier than recommended (age unspecified)	*Introduction of cow's milk < 12 months*:<br>- Lower maternal education and knowledge in appropriate CF practices, multiparity, not having food allergies/ family history of atopies
	Poland, n= 251, predominantly mothers with a university degree, and ~ half adhering to vegetarianism [34]	10% of infants whose mothers were vegetarian were also vegetarian. Vegetarian mothers started CF with vegetables and fruits. Meats were introduced at ~ 5.3 months in omnivore mothers (later in vegetarians)	*Earlier introduction of cow's milk*:<br>- Maternal diet being omnivore<br>*Later introduction of meat*:<br>- Maternal diet being vegetarian
	Spain, n = 630, predominantly married/partnered mothers with a university degree [78]	78% started CF with iron-fortified cereals (median: 5 months), 46% of 7-11 months' infants consumed them every day. Meats were introduced ~ 7 months (median) and offered ~ 3-4 times/week in 7-11 months infants.	-
	United Kingdom, n = 2747, national survey conducted in Scotland [36]	At 8-12 months, 3% reported giving tea and 4% cow's milk to their infants	-
	United Kingdom, n = 134, predominantly mothers of White ethnicity with a university degree [37]	Iron-fortified cereals and meat/fish were offered < once/day at 6-8 months. Total daily iron intake was 6.3mg (TSF) and 4.2mg (BLW) [6].	- *Higher consumption of iron-fortified cereals at 6-8 months*: TSF
	United Kingdom, n = 64, mostly highly educated although ~ half living in deprived areas [38]	94% of 4-12 months infants did not consume cow's milk. 44% offered meat, fish or eggs ≤ once/week,	- *Offering meat, fish or eggs ⩾ once/week*: Living in a less deprived area

		although 23% offered them ≥ 7 times/week	
	United Kingdom, n = 565, > half of the sample occupying managerial positions [39]	110 parents (on 567) started CF with iron-fortified cereals and 12 parents with meat	- *Higher consumption of iron-fortified cereals at the beginning of CF*: TSF
	United Kingdom, n = 96, highly educated with high proportion having managerial work positions [44]	44 % (TSF) and 63% (BLW) of infants aged 6-8 months consumed < 4.2mg of iron/day in a 24h recall	- *Lower consumption of iron-fortified cereals at 6-8 months:* BLW
**Oceania**	Australia, n = 828, comparable to the maternal population of South Australia; mostly born in Australia/New Zealand and more than half highly educated [90]	Major food sources of iron in infants aged 12-14 months: cereal-based and commercial infant foods (such as iron-fortified cereals). Meat only contributed to ~ 6% of daily iron intake. Daily iron intake was ~ 7mg (24% were ≥ 9mg (RDI) and 21% < 4mg (EAR))	- *Lower iron intake*: Breastfeeding
	Australia, n = 4981, high maternal education level [48]	75% started CF with cereals (not specified whether iron-fortified or not)	-
	New Zealand, n = 876, highly educated [51]	48% consumed iron-fortified cereals at 6 months and 56% red meats	- *Lower consumption of iron-fortified cereals*: BLW<br>- *Higher consumption of red meat:* BLW
**Asia**	Bangladesh, n = 2167 (2017-2018), nationally representative [53]	In a 24h recall, 67% of infants aged 6-23 months consumed meat, fish or eggs (increase as compared to past national surveys) and 22% legumes and nuts (decrease as compared to 2007).	-
	Cambodia (n = 222), Nepal (n=228), Senegal (n = 218) and Tanzania (n = 229) predominantly married mothers with varying socio-economic status [81]	In a 24h recall, 22% of 6-11 months' Tanzanian infants drank tea/coffee and 15% of Nepali infants. Iron-fortified cereals were not consumed often (highest: ~ 30% in Senegal and Nepal). Meat and seafood consumption < 12 months was only high in Cambodia (74%)	-
	China, n = 760, mostly highly educated employed mothers (~ half of them intending to come back to work ≤ 6 months post-partum) [56]	78% introduced iron-fortified cereals ≤ 6 months and 53% introduced meat/eggs ≤ 6 months	-
	China, n = 89006 (2015), nationally representative [79]	Meat introduction: 8.7 months (urban), 9.3 months (suburban)	-
	China, n = 2251, predominantly mothers of Han Chinese ethnicity, > half with ≥ 16 years of education [58]	At 6 months, 94% introduced cereals [7] and 2% meats	-
	India, n = 902, mostly stay-at-home mothers with secondary education [8] [60]	22% of infants aged 12 months consumed eggs/flesh foods (24h recall)	-

	Lebanon, n = 1051, representative of Lebanese children enrolled in daycare centers, mostly highly educated married parents [61]	54% started CF with iron-fortified cereals. 57% consumed tea < 4 months. Meats were introduced ~ 9 months (median = 8 months)	-
	Malaysia, n = 119, Malay mothers residing in an urban area [80]	62% of infants aged 6-11 months consumed commercial baby foods (including iron-fortified cereals) and 27% meats. Daily iron intake from foods was 6.2 mg (69% RNI)	-
	Nepal, n = unspecified (2016), nationally representative [62]	39% of infants aged 6-23 months consumed iron-rich foods (undefined) and 42% animal products (dairy, eggs and flesh foods) (increased compared to past surveys)	-
	Sri Lanka, n = 515, mostly residing in rural areas, predominantly identifying as housewives [65]	Infant cereals were not commonly used as a first CF (3%) and 31% of 6-12 months infants had consumed tea	-
	Thailand, n = 108, mostly middle-class, highly educated mother or family member [67]	Parents started CF with porridge and mashed fruits; ~13% introduced cow's milk < 12 months	-
	United Arab Emirates, n = 276, ~ half of parents with university degrees [69]	Daily iron intake was 10.3mg (total) in infants aged 6-11 months, although 47% had intakes < 6.9mg/day (EAR) in a 24h recall	-
**Africa**	Ethiopia, n = 323, mothers of Oromo ethnicity, mostly married, living in rural areas and with low education levels [72]	Flesh foods were not common during the CF period; most children consumed legumes and nuts, but grains, roots and tubers were predominant	-
	Senegal, n = 98, low education, high prevalence of food insecurity [82]	49% of children aged 6-11 months consumed iron-rich foods (fish, eggs, meat and iron-fortified foods) (24h recall)	- *Lower consumption of iron-rich foods:* Household food insecurity
	Zanzibar, Tanzania, n = 200 [75]	65% of 6-23 months infants consumed sweetened tea. Daily iron intake from foods (24h recall) in 6-11 months infants was 1.74 mg	-

[1] CF: Complementary Feeding

[2] WIC: Special Supplemental Nutrition Program for Women, Infants, and Children (WIC)

[3] Including infant formulas and breastmilk

[4] EFF: Exclusive formula-feeding; EBF: Exclusive breastfeeding

[5] The proportion of mothers with ≥ 12 years of education increased in the 2015 cohort as compared to 2004 (31 vs 10%)

[6] TSF: Traditional spoon-feeding; BLW: Baby-led weaning. In this study, the Lower Reference Nutrient Intake for iron was 4.2mg/day

[7] Not specified if this includes iron-fortified infant cereals

[8] Participants had already undergone a RCT during pregnancy and postpartum which assessed the effect of DHA supplementation on child neurodevelopment

Table S5. Detailed consumption of added sugars, juice, and honey during the CF [1] period across continents.

Continent	Setting (country, n, sample characteristics)	Consumption of Sugar during the CF Period	Factors Associated with Added Sugar Consumption
North America	Canada, n = 22, Middle Eastern mothers, low socio-economic status [1]	Sweetened water was among the first complementary foods even < 4 months	-
	United States, n = 3777, nationally representative sample of mothers enrolled in the WIC federal program [2] [5]	~25% consumed sweet foods ≤ 8 months and ~ 60% < 12 months (decrease compared to the first WIC cohort). SSB's [3]: 11% < 6 months (median: 12.6 months). 100% fruit juice: 50% < 7 months (median: 6.9 months).	- Non-Hispanic/Latino race/ethnicity<br>- Born in the United States<br>- Younger maternal age<br>- Mother unmarried
	United States, n = 1261, nationally representative sample of mothers enrolled in the WIC federal program [83]	31% of children were exposed to sweet drinks (including 100% juice) ≤ 7 months, only 14% waited > 12 months.	-
	United States, n = 3235, more educated and higher proportion of White Non-Hispanic parents than US population [6]	6-12 months: 27% offered 100% fruit juice and 34% UPF [4] including 8.5% SSBs (24h recall)	- Non-Hispanic black race/ethnicity (in toddlers)
	United States, n = 449, mostly low-income, non-Hispanic Black mothers [7]	71% consumed > 1 UPF/day < 12 months (including sweets) although not < 4 months	-
	United States, n = 1211, nationally representative sample [91]	61% of 6-11 months infants consumed added sugars (24h recall) (~ 1 tsp/day), mainly from yogurt, commercial infant snacks/sweets and bakery products	- Non-Hispanic Black race/ethnicity
	United States, n = 443, low income/education, mostly of Non-Hispanic White and Black ethnicity/race [10]	20% consumed juice and other beverages ≤ 6 months, none reported SSB's	-
	United States, n = 217, highly educated, high income, mostly non-Hispanic White mothers. [12]	Introduction of sweet foods/beverages ~ 8 months and 93% < 12 months	-
South America	Brazil, n = 1567, low education, 1/3 having ≤ minimum wage [14]	36% introduced sugar < 4 months and 8% honey	- Lower maternal education<br>- Lower family income
	Brazil, n = 700, high prevalence of FF [5] [87]	Median age for juice introduction was 4 months (68%)	-
	Brazil, n = 79, low income and education [15]	14% started CF with fruit juice and 13% offered it < 6 months. Almost all children aged 6-24 months consumed sugar rich UPF	-
	Brazil, n = 545, mostly of White or Multiracial/Asian ethnicity, low education [92]	74% of 6-12 months infants consumed breakfast cereals, 46% sweetened cheese/yogurt, 28% candies/chocolate milk, 17% SSBs	- Having < 4 people in the household<br>- FF
	Brazil, n = 3823 (2004); n = 3689 (2015) [16]	Decrease in the consumption of juice < 6 months (68% in 2004 vs 43% in 2015).	-

		~ 20% consumed soft drinks < 12 months in 2015	
	Brazil, n = 847, mostly mothers of Black or Brown race/ethnicity, low education [88]	18% of infants aged < 6 months consumed foods containing added sugars, 17% cookies or crackers and 5% juices (24h recall)	- Lower BF [6]
	Brazil, n = 599, mothers with low levels of education [93]	79% of children aged 6-24 months consumed UPF including 35% aged 6-12 months.	- Beneficiation of social government programs<br>- Living with 4+ people
	Brazil, n = 231, mostly educated low-middle class mothers [94]	94% consumed processed and UPF during CF	- FF
	Brazil, n = 538, mothers of Black or Brown race/ethnicity [95]	Between 6-12 months, 30% of daily energy intake came from UPF	-
	Brazil, n = 300, mostly multiparous mothers with low-middle education and average income [96]	≤ 6 months, 27% of children consumed jello, 24% sweetened cheese/yogurt and 20% to cookies	- Lower maternal education<br>- Lower income<br>- Multiparity<br>- Older maternal age
	Chile, n = 261, ~ half with > 16 years of education [17]	Among infants aged < 24 months, 9% consumed added sugars in their food, 8% honey, 20% were introduced to juices (~ 1.6 serving/day) and 56% cookies	-
	Haiti, n = 310, low education and income [97]	~ ¼ of infants consumed liquids including *maje kaan*, a sweetened traditional drink, in the first days	-
	Mexico, n = 949, nationally representative survey [98]	35% of 6-12 months consumed sweetened milks, 13% fruit juice, 9% fruit flavored drinks, 7% sodas, 10% sweetened tea/coffee and 17% traditional drinks (sweetened) (24h recall)	- Lower education
	Mexico, n = 143, low socio-economic status [20]	55% of infants consumed juice at 6 months.	-
**Europe**	Europe (Belgium, Bulgaria, Germany, Greece, Poland and Spain) n = 6800 [21]	Fruit juices were introduced at a median age of 6 months (Bulgaria most early)	-
	France, n = 10907, predominantly born in France, and a little less than half having ≥ 15 years of education [99]	9% of parents added sugars to foods at 6 months, this increased to 25% at 10 months	- BF<br>- Very early CF (< 4 months)<br>- Relying on personal experience/family for information
	Poland, n = 4110 and Austria, n = 1783, mothers of children aged 12-36 months, highly educated (more so in Poland than in Austria) [85]	33% of infants aged 12-24 months consumed added sugars	- Multiparity<br>- Younger maternal age<br>- Living in rural areas<br>- Lower maternal income (in Poland)

			- Not currently BF (in Austria) and lower BF duration (in Poland)
	United Kingdom, n = 1327, multi-ethnic cohort [23]	50% started CF with a sweetened food and 10% consumed sweetened drinks < 4 months	- Pakistani and Other South Asian race/ethnicity
	United Kingdom, n = 2747, national survey conducted in Scotland [36]	29% of parents offered UPF (chocolate, ice cream, snacks, etc.) to 8-12 months infants	- Younger maternal age<br>- Living in a more deprived area
	United Kingdom, n = 134, predominantly mothers of White race/ethnicity with university degrees [37]	Sugary foods were offered < than once per day at 6-12 months.	- TSF (in 6-8 months infants) [7]
	United Kingdom, n = 64, most highly educated although ~ half living in deprived areas [38]	64% of parents did not offer sweet foods (< 12 months), although 20% offered them 2-4 times per week	- Living in a less deprived area
	United Kingdom, n = 110, multi-ethnic cohort living in a more deprived town [41]	Consumption of *gutti* (containing honey) during CF in Pakistani/Bangladeshi culture	-
	United Kingdom, n = 30 mothers + 30 fathers, ~ half with an undergraduate degree [42]	67% fathers and 77% mothers avoided sweet drinks during CF, 48-60% offered confectionaries (sweets) once-thrice weekly	-
**Oceania**	Australia, n = 828, comparable to the maternal population of South Australia [46]	96% of infants aged ≤ 24 months were offered UPF during the 3 days recalled (including 37% cookies, 15% pastries and 16% sugar/honey/syrups)	- Younger maternal age<br>- Primiparity<br>- Maternal country of birth (Australia and the UK as compared to Asian countries)
**Asia**	Cambodia (n = 222), Nepal (n=228), Senegal (218) and Tanzania (229) predominantly married mothers with varying socio-economic status [81]	In a 24h recall, 27-31% of 12-23 months infants from Senegal and Tanzania consumed juice. 70% of 6-11 months Tanzanian infants consumed sugar or honey. 58% (6-11 months) and 83% (12-23 months) of Nepali infants had consumed UPF such as pastries, candies and salty snacks.	- Lower maternal education (in Senegal)<br>- Lower economic status (in Cambodia)<br>- Not offering breastmilk substitute (in Senegal)
	China, n = 408, mostly ≤ senior middle school education level, around half of them unemployed [57]	74% of children aged 6-24 months consumed UPF (55% pastries, 15% confectioneries, 13% SSB's, etc.)	- Parental/caregiver emotional and instrumental feeding
	China, n = 2251, predominantly mothers of Han Chinese ethnicity, > half of them with ≥ 16 years of education [58]	At 12 months, 88% of children had been introduced to sweets	-
	India, n = 297, married mothers from the Santal tribe with low education [59]	31% started CF with "biscuits"	-
	India, n = 902, mostly stay-at-home mothers with a secondary education level [7] [60]	Prevalence of pre-lacteal feed (sweetened water, honey, etc.): 9%. 5%	-

		of 12 months infants consumed SSB's and 86% UPF	
	Indonesia, n = 495, most mothers had a secondary education level and were wealthier as compared to the 2012 national survey [100]	47% of infants aged 6-11 months consumed UPF, and 85% of 12-17 months' infants. Almost no infants consumed SSB's (sweetened milk and tea) < 12 months but 50% at 18-23 months.	- Lower maternal education<br>- No offering of commercial infant foods<br>- No offering of breastmilk substitute
	Lebanon, n = 1051, representative of Lebanese children enrolled in daycare centers, mostly highly educated married parents [61]	50% introduced cookies and 25% milk-based desserts at the beginning of CF.	-
	Malaysia, n = 119, Malay mothers residing in an urban area [80]	34% of infants aged < 24 months consumed sweetened desserts, 6% SSB's, 24% *Kuih* (traditional cake)	-
	Nepal, n = 309, predominantly married [86]	52% of infants aged < 24 months consumed added sweeteners in their foods/drinks (24h recall). 85% consumed cookies and 64% candies/chocolate the last week.	-
	South India, n = 95, highly educated, mostly middle-class mothers identifying as housewives [64]	81% mentioned adding sugar to complementary foods (infants aged 12-24 months)	-
	Sri Lanka, n = 515, mostly identifying as housewives and residing in rural areas [65]	Salt and sugar were introduced at a mean age of 7.6 months, 38% of children aged 6-12 months had been introduced to chocolate.	-
	Thailand, n = 108, mostly middle-class, highly educated mother or family member [67]	26% of infants aged 6-8 months consumed 1-2 oz of fruit juice/day. 13% added salt and sugar at 6-8 months, and 48% at 13-18 months.	-
	United Arab Emirates, n = 1822, mostly married, highly educated [68]	52% of infants aged 6-24 months drank SSB's or tea once-twice daily	-
	United Arab Emirates, n = 276, slightly more than half of parents highly educated [69]	In a 24h recall, infants aged 6-12 months consumed ~ 5g of added sugars/day. This increased to ~ 14g in the 12-24 months' group. 57% of 12-23 months children consumed > 5% of their daily energy in sugar	-
**Africa**	Zanzibar, Tanzania, n = 200 [75]	65% of infants aged 6-23 months consumed sweetened tea	-

[1] CF: Complementary Feeding.
[2] WIC: Special Supplemental Nutrition Program for Women, Infants, and Children (WIC).
[3] SSB's: Sugar-sweetened beverages.
[3] UPF: Ultra-processed foods, typically high in added sugars, salt and fat.
[4] FF: Formula-feeding.
[5] BF: Breastfeeding.
[6] TSF: Traditional spoon-feeding method to CF.

[7] Participants had already undergone a RCT during pregnancy and postpartum which assessed the effect of DHA supplementation on child neurodevelopment.

References

1. Jessri, M.; Farmer, A.P.; Olson, K. A focused ethnographic assessment of Middle Eastern mothers' infant feeding practices in Canada. *Matern Child Nutr* **2015**, *11*, 673-686, doi:10.1111/mcn.12048.
2. Differding, M.K.; Doyon, M.; Bouchard, L.; Perron, P.; Guérin, R.; Asselin, C.; Massé, E.; Hivert, M.F.; Mueller, N.T. Potential interaction between timing of infant complementary feeding and breastfeeding duration in determination of early childhood gut microbiota composition and BMI. *Pediatr Obes* **2020**, *15*, e12642, doi:10.1111/ijpo.12642.
3. Fialkowski, M.K.; Ng-Osorio, J.; Kai, J.; Swafford, K.; Langfelder, G.; Young, C.G.; Chen, J.J.; Zhu, F.M.; Boushey, C.J. Type, Timing, and Diversity of Complementary Foods Among Native Hawaiian, Pacific Islander, and Filipino Infants. *Hawaii J Health Soc Welf* **2020**, *79*, 127-134.
4. Doub, A.E.; Moding, K.J.; Stifter, C.A. Infant and maternal predictors of early life feeding decisions. The timing of solid food introduction. *Appetite* **2015**, *92*, 261-268, doi:10.1016/j.appet.2015.05.028.
5. May;, L.; Borger;, C.; Weinfield;, N.; MacAllum;, C.; DeMatteis;, J.; McNutt;, S.; Whaley;, S.; Ritchie;, L.; Sallack;, L. *WIC Infant and Toddler Feeding Practices Study – 2: Infant Year Report;* US Department of Agriculture: Alexandria, VA, USA, 2017.
6. Roess, A.A.; Jacquier, E.F.; Catellier, D.J.; Carvalho, R.; Lutes, A.C.; Anater, A.S.; Dietz, W.H. Food Consumption Patterns of Infants and Toddlers: Findings from the Feeding Infants and Toddlers Study (FITS) 2016. *J Nutr* **2018**, *148*, 1525s-1535s, doi:10.1093/jn/nxy171.
7. Vadiveloo, M.; Tovar, A.; Østbye, T.; Benjamin-Neelon, S.E. Associations between timing and quality of solid food introduction with infant weight-for-length z-scores at 12 months: Findings from the Nurture cohort. *Appetite* **2019**, *141*, 104299, doi:10.1016/j.appet.2019.05.030.
8. Chiang, K.V.; Hamner, H.C.; Li, R.; Perrine, C.G. Timing of Introduction of Complementary Foods - United States, 2016-2018. *MMWR Morb Mortal Wkly Rep* **2020**, *69*, 1787-1791, doi:10.15585/mmwr.mm6947a4.
9. Chaparro, M.P.; Anderson, C.E. Differences in Early Childhood Dietary Behaviors by Infant Feeding Type and Sex. *J Nutr* **2021**, *151*, 2001-2009, doi:10.1093/jn/nxab076.
10. Hornsby, P.P.; Conaway, M.R.; Medico, T.J.; Gurka, K.K.; Kellams, A. Timing of Introduction of Complementary Foods and Beverages to Infants of Low-Income Women. *Breastfeed Med* **2021**, *16*, 547-552, doi:10.1089/bfm.2020.0352.
11. Kong, K.L.; Burgess, B.; Morris, K.S.; Re, T.; Hull, H.R.; Sullivan, D.K.; Paluch, R.A. Association Between Added Sugars from Infant Formulas and Rapid Weight Gain in US Infants and Toddlers. *J Nutr* **2021**, *151*, 1572-1580, doi:10.1093/jn/nxab044.
12. Sanjeevi, N.; Lipsky, L.M.; Siega-Riz, A.M.; Nansel, T.R. Associations of infant appetitive traits during milk feeding stage with age at introduction to solids and sweet food/beverage intake. *Appetite* **2022**, *168*, 105669, doi:https://doi.org/10.1016/j.appet.2021.105669.
13. Surette, V.A.; Smith-Simpson, S.; Fries, L.R.; Ross, C.F. Food texture experiences across age groups in 4- to 36-month-old children in the United States. *J Texture Stud* **2022**, *53*, 18-30, doi:10.1111/jtxs.12649.
14. Dallazen, C.; Silva, S.A.D.; Gonçalves, V.S.S.; Nilson, E.A.F.; Crispim, S.P.; Lang, R.M.F.; Moreira, J.D.; Tietzmann, D.C.; Vítolo, M.R. Introduction of inappropriate complementary feeding in the first year of life and associated factors in children with low socioeconomic status. *Cad Saude Publica* **2018**, *34*, e00202816, doi:10.1590/0102-311x00202816.
15. Neves, A.M.; Madruga, S.W. Complementary feeding, consumption of industrialized foods and nutritional status of children under 3 years old in Pelotas, Rio Grande do Sul, Brazil, 2016: a descriptive study. *Epidemiol Serv Saude* **2019**, *28*, e2017507, doi:10.5123/s1679-49742019000100019.
16. Schneider, B.C.; Gatica-Domínguez, G.; Assunção, M.C.F.; Matijasevich, A.; Barros, A.J.D.; Santos, I.S.; Silveira, M.F. Introduction to complementary feeding in the first year of life and risk of overweight at 24 months of age: changes from 2004 to 2015 Pelotas (Brazil) Birth Cohorts. *Br J Nutr* **2020**, *124*, 620-630, doi:10.1017/s0007114520001634.
17. Quintiliano-Scarpelli, D.; Lehmann, N.; Castillo, B.; Blanco, E. Infant Feeding and Information Sources in Chilean Families Who Reported Baby-Led Weaning as a Complementary Feeding Method. *Nutrients* **2021**, *13*, 2707. doi:10.3390/nu13082707.

18. Sepúlveda Alarcón, N.; Le Roy Olivos, C. Dietary intake of protein sources in infants between 7-24 months old: Start time and compliance with recommendations. *Andes Pediatr* **2021**, *92*, 79-85, doi:10.32641/andespediatr.v92i1.2308.
19. Ortega-Cisneros, C.M.; Vidaña-Pérez, D.; Basto-Abreu, A.; Iglesias-Leboreiro, J.; Venegas-Andrade, A.; Rodriguez-Santaolaya, P.; López-Arzat, L.V.; Blanco-Montero, A. Complementary feeding practices in Mexican healthy infants: How close are they to the current guidelines? *Bol Med Hosp Infant Mex* **2019**, *76*, 265-272, doi:10.24875/bmhim.19000064.
20. Maas-Mendoza, E.; Vega-Sánchez, R.; Vázquez-Osorio, I.M.; Heller-Rouassant, S.; Flores-Quijano, M.E. Infant Feeding Practices That Substitute Exclusive Breastfeeding in a Semi-Rural Mexican Community: Types, Moments, and Associated Factors. *Nutrients* **2022**, *14*, 2017. doi:10.3390/nu14102017.
21. Usheva, N.; Galcheva, S.; Cardon, G.; De Craemer, M.; Androutsos, O.; Kotowska, A.; Socha, P.; Koletzko, B.V.; Moreno, L.A.; Iotova, V., et al. Complementary Feeding and Overweight in European Preschoolers: The ToyBox-Study. *Nutrients* **2021**, *13*, 1199. doi:10.3390/nu13041199.
22. Kronborg, H.; Foverskov, E.; Væth, M. Predictors for early introduction of solid food among Danish mothers and infants: an observational study. *BMC Pediatr* **2014**, *14*, 243, doi:10.1186/1471-2431-14-243.
23. Santorelli, G.; Fairley, L.; Petherick, E.S.; Cabieses, B.; Sahota, P. Ethnic differences in infant feeding practices and their relationship with BMI at 3 years of age - results from the Born in Bradford birth cohort study. *Br J Nutr* **2014**, *111*, 1891-1897, doi:10.1017/s0007114514000099.
24. Bournez, M.; Ksiazek, E.; Wagner, S.; Kersuzan, C.; Tichit, C.; Gojard, S.; Thierry, X.; Charles, M.A.; Lioret, S.; de Lauzon-Guillain, B., et al. Factors associated with the introduction of complementary feeding in the French ELFE cohort study. *Matern Child Nutr* **2018**, *14*, e12536, doi:10.1111/mcn.12536.
25. Chouraqui, J.P.; Tavoularis, G.; Emery, Y.; Francou, A.; Hébel, P.; Bocquet, M.; Hankard, R.; Turck, D. The French national survey on food consumption of children under 3 years of age - Nutri-Bébé 2013: design, methodology, population sampling and feeding practices. *Public Health Nutr* **2018**, *21*, 502-514, doi:10.1017/s1368980017002518.
26. Demonteil, L.; Ksiazek, E.; Marduel, A.; Dusoulier, M.; Weenen, H.; Tournier, C.; Nicklaus, S. Patterns and predictors of food texture introduction in French children aged 4-36 months. *Br J Nutr* **2018**, *120*, 1065-1077, doi:10.1017/s0007114518002386.
27. Marduel Boulanger, A.; Vernet, M. Introduction of new food textures during complementary feeding: Observations in France. *Arch Pediatr* **2018**, *25*, 6-12, doi:10.1016/j.arcped.2017.10.025.
28. Baldassarre, M.E.; Panza, R.; Farella, I.; Posa, D.; Capozza, M.; Mauro, A.D.; Laforgia, N. Vegetarian and Vegan Weaning of the Infant: How Common and How Evidence-Based? A Population-Based Survey and Narrative Review. *Int J Environ Res Public Health* **2020**, *17*, 4835. doi:10.3390/ijerph17134835.
29. Addessi, E.; Galloway, A.T.; Wingrove, T.; Brochu, H.; Pierantozzi, A.; Bellagamba, F.; Farrow, C.V. Baby-led weaning in Italy and potential implications for infant development. *Appetite* **2021**, *164*, 105286, doi:10.1016/j.appet.2021.105286.
30. Alvisi, P.; Congiu, M.; Ficara, M.; De Gregorio, P.; Ghio, R.; Spisni, E.; Di Saverio, P.; Labriola, F.; Lacorte, D.; Lionetti, P. Complementary Feeding in Italy: From Tradition to Innovation. *Children (Basel)* **2021**, *8*, 638. doi:10.3390/children8080638.
31. Helle, C.; Hillesund, E.R.; Øverby, N.C. Timing of complementary feeding and associations with maternal and infant characteristics: A Norwegian cross-sectional study. *PLoS One* **2018**, *13*, e0199455, doi:10.1371/journal.pone.0199455.
32. Zielinska, M.A.; Rust, P.; Masztalerz-Kozubek, D.; Bichler, J.; Hamułka, J. Factors Influencing the Age of Complementary Feeding-A Cross-Sectional Study from Two European Countries. *Int J Environ Res Public Health* **2019**, *16*, 3799. doi:10.3390/ijerph16203799.
33. Kostecka, M.; Jackowska, I.; Kostecka, J. Factors Affecting Complementary Feeding of Infants. A Pilot Study Conducted after the Introduction of New Infant Feeding Guidelines in Poland. *Nutrients* **2020**, *13*, 61. doi:10.3390/nu13010061.
34. Kostecka, M.; Kostecka-Jarecka, J. Knowledge on the Complementary Feeding of Infants Older than Six Months among Mothers Following Vegetarian and Traditional Diets. *Nutrients* **2021**, *13*, 3973. doi:10.3390/nu13113973.
35. Brown, A. Differences in eating behaviour, well-being and personality between mothers following baby-led vs. traditional weaning styles. *Matern Child Nutr* **2016**, *12*, 826-837, doi:10.1111/mcn.12172.

36. Scottish Government. Scottish maternal and infant nutrition survey 2017. Availabe online: https://www.gov.scot/publications/scottish-maternal-infant-nutrition-survey-2017/ (accessed on 10 March 2023).
37. Alpers, B.; Blackwell, V.; Clegg, M.E. Standard v. baby-led complementary feeding: a comparison of food and nutrient intakes in 6-12-month-old infants in the UK. *Public Health Nutr* **2019**, *22*, 2813-2822, doi:10.1017/s136898001900082x.
38. Garcia, A.L.; Looby, S.; McLean-Guthrie, K.; Parrett, A. An Exploration of Complementary Feeding Practices, Information Needs and Sources. *Int J Environ Res Public Health* **2019**, *16*, 4311. doi:10.3390/ijerph16224311.
39. Komninou, S.; Halford, J.C.G.; Harrold, J.A. Differences in parental feeding styles and practices and toddler eating behaviour across complementary feeding methods: Managing expectations through consideration of effect size. *Appetite* **2019**, *137*, 198-206, doi:10.1016/j.appet.2019.03.001.
40. Rogers, S.L.; Blissett, J. Infant temperament, maternal feeding behaviours and the timing of solid food introduction. *Matern Child Nutr* **2019**, *15*, e12771, doi:10.1111/mcn.12771.
41. Cook, E.J.; Powell, F.C.; Ali, N.; Penn-Jones, C.; Ochieng, B.; Randhawa, G. Parents' experiences of complementary feeding among a United Kingdom culturally diverse and deprived community. *Matern Child Nutr* **2021**, *17*, e13108, doi:10.1111/mcn.13108.
42. Spyreli, E.; McKinley, M.C.; Dean, M. Comparing Maternal and Paternal Complementary Feeding Practices: Findings From an Online Survey. *J Nutr Educ Behav* **2021**, *53*, 531-539, doi:10.1016/j.jneb.2020.11.018.
43. Webber, C.; Blissett, J.; Addessi, E.; Galloway, A.T.; Shapiro, L.; Farrow, C. An infant-led approach to complementary feeding is positively associated with language development. *Matern Child Nutr* **2021**, *17*, e13206, doi:10.1111/mcn.13206.
44. Pearce, J.; Langley-Evans, S.C. Comparison of food and nutrient intake in infants aged 6-12 months, following baby-led or traditional weaning: A cross-sectional study. *J Hum Nutr Diet* **2022**, *35*, 310-324, doi:10.1111/jhn.12947.
45. Bell, L.K.; Jansen, E.; Mallan, K.; Magarey, A.M.; Daniels, L. Poor dietary patterns at 1-5 years of age are related to food neophobia and breastfeeding duration but not age of introduction to solids in a relatively advantaged sample. *Eat Behav* **2018**, *31*, 28-34, doi:10.1016/j.eatbeh.2018.06.005.
46. Coxon, C.; Devenish, G.; Ha, D.; Do, L.; Scott, J.A. Sources and Determinants of Discretionary Food Intake in a Cohort of Australian Children Aged 12-14 Months. *Int J Environ Res Public Health* **2019**, *17*, 80. doi:10.3390/ijerph17010080.
47. Arora, A.; Manohar, N.; Hector, D.; Bhole, S.; Hayen, A.; Eastwood, J.; Scott, J.A. Determinants for early introduction of complementary foods in Australian infants: findings from the HSHK birth cohort study. *Nutr J* **2020**, *19*, 16, doi:10.1186/s12937-020-0528-1.
48. Moss, K.M.; Dobson, A.J.; Tooth, L.; Mishra, G.D. Associations between feeding practices in infancy and fruit and vegetable consumption in childhood. *Br J Nutr* **2020**, *124*, 1320-1328, doi:10.1017/s000711452000238x.
49. Australian Bureau of Statistics. Breastfeeding. Availabe online: https://www.abs.gov.au/statistics/health/health-conditions-and-risks/breastfeeding/latest-release (accessed on 10 March 2023).
50. Netting, M.J.; Moumin, N.A.; Knight, E.J.; Golley, R.K.; Makrides, M.; Green, T.J. The Australian Feeding Infants and Toddler Study (OzFITS 2021): Breastfeeding and Early Feeding Practices. *Nutrients* **2022**, *14*, 206. doi:10.3390/nu14010206.
51. Fu, X.; Conlon, C.A.; Haszard, J.J.; Beck, K.L.; von Hurst, P.R.; Taylor, R.W.; Heath, A.M. Food fussiness and early feeding characteristics of infants following Baby-Led Weaning and traditional spoon-feeding in New Zealand: An internet survey. *Appetite* **2018**, *130*, 110-116, doi:10.1016/j.appet.2018.07.033.
52. Al Mamun, M.A.; Saha, S.; Li, J.; Binta, A.G.R.; Al Hasan, S.M.; Begum, A. Child Feeding Practices of Childbearing Mothers and Their Household Food Insecurity in a Coastal Region of Bangladesh. *Inquiry* **2022**, *59*, 469580221096277, doi:10.1177/00469580221096277.
53. Mistry, S.K.; Hossain, M.B.; Irfan, N.M.; Saha, M.; Saberin, S.; Shamim, A.A.; Arora, A. Trends in Complementary Feeding Indicators and Intake from Specific Food Groups among Children Aged 6-23 Months in Bangladesh. *Int J Environ Res Public Health* **2022**, *19*, 550. doi:10.3390/ijerph19010550.
54. Wang, F.; Liu, H.; Wan, Y.; Li, J.; Chen, Y.; Zheng, J.; Huang, T.; Li, D. Age of Complementary Foods Introduction and Risk of Anemia in Children Aged 4-6 years: A Prospective Birth Cohort in China. *Sci Rep* **2017**, *7*, 44726, doi:10.1038/srep44726.

55. Liu, X.; Liao, X.; Ren, Q.; Luo, M.; Yang, L.; Lin, J.; Chang, J. Concerns regarding complementary feeding practices among urban Chinese mothers: a focus group study in Xi'an. *J Health Popul Nutr* **2018**, *37*, 20, doi:10.1186/s41043-018-0151-3.
56. Yu, C.; Binns, C.W.; Lee, A.H. The Early Introduction of Complementary (Solid) Foods: A Prospective Cohort Study of Infants in Chengdu, China. *Nutrients* **2019**, *11*, 760. doi:10.3390/nu11040760.
57. An, M.; Liu, X.; Guo, H.; Zhou, Q. The Associations between Caregivers' Emotional and Instrumental Feeding, Children's Emotional Eating, and Children's Consumption of Ultra-Processed Foods in China. *Int J Environ Res Public Health* **2022**, *19*, 4439. doi:10.3390/ijerph19084439.
58. Zhong, C.; Guo, J.; Tan, T.; Wang, H.; Lin, L.; Gao, D.; Li, Q.; Sun, G.; Xiong, G.; Yang, X., et al. Increased food diversity in the first year of life is inversely associated with allergic outcomes in the second year. *Pediatr Allergy Immunol* **2022**, *33*, e13707, doi:10.1111/pai.13707.
59. Stiller, C.K.; Golembiewski, S.K.E.; Golembiewski, M.; Mondal, S.; Biesalski, H.K.; Scherbaum, V. Maternal nutritional status and child feeding practices: a retrospective study in Santal communities, Birbhum District, West Bengal, India. *Int Breastfeed J* **2020**, *15*, 50, doi:10.1186/s13006-020-00262-3.
60. Khandelwal, S.; Kondal, D.; Chakravarti, A.R.; Dutta, S.; Banerjee, B.; Chaudhry, M.; Patil, K.; Swamy, M.K.; Ramakrishnan, U.; Prabhakaran, D., et al. Infant Young Child Feeding Practices in an Indian Maternal-Child Birth Cohort in Belagavi, Karnataka. *Int J Environ Res Public Health* **2022**, *19*, 5088. doi:10.3390/ijerph19095088.
61. Issa, C.; Hobeika, M.; Khairallah, W.; Al-Jawaldeh, A.; Batal, M. Timing and types of fluids and foods first introduced in a representative sample of toddlers attending day care programs across Lebanon: Are parents following international recommendations? *J Pediatr Nurs* **2022**, *62*, e45-e53, doi:10.1016/j.pedn.2021.07.003.
62. Hanley-Cook, G.; Argaw, A.; Dahal, P.; Chitekwe, S.; Kolsteren, P. Infant and young child feeding practices and child linear growth in Nepal: Regression-decomposition analysis of national survey data, 1996-2016. *Matern Child Nutr* **2022**, *18 Suppl 1*, e12911, doi:10.1111/mcn.12911.
63. Duggal, M.N.; Bari, A.; Iftikhar, A. Complementary feeding practices among mothers of children aged six months to two years at Children's hospital Lahore. *J Pak Med Assoc* **2020**, *70*, 1543-1546, doi:10.5455/jpma.24349.
64. Ganesan, S.; Jayaraj, J.; Geminiganesan, S.; Rajan, M. A study on parental awareness of feeding practices in children in the age-group 12-24 months. *J Prev Med Hyg* **2021**, *62*, E909-e917, doi:10.15167/2421-4248/jpmh2021.62.4.2287.
65. Bandara, T.; Hettiarachchi, M.; Liyanage, C.; Amarasena, S. Current infant feeding practices and impact on growth in babies during the second half of infancy. *J Hum Nutr Diet* **2015**, *28*, 366-374, doi:10.1111/jhn.12253.
66. Hua, M.-C.; Yao, T.-C.; Chen, C.-C.; Tsai, M.-H.; Liao, S.-L.; Lai, S.-H.; Chiu, C.-Y.; Su, K.-W.; Yeh, K.-W.; Huang, J.-L. Introduction of various allergenic foods during infancy reduces risk of IgE sensitization at 12 months of age: a birth cohort study. *Pediatric Research* **2017**, *82*, 733-740, doi:10.1038/pr.2017.174.
67. Kittisakmontri, K.; Fewtrell, M.; Roekworachai, K.; Phanpong, C.; Lanigan, J. Complementary feeding: Attitudes, knowledge and practices of urban families in northern Thailand. *Nutr Diet* **2019**, *76*, 57-66, doi:10.1111/1747-0080.12474.
68. Taha, Z.; Garemo, M.; Nanda, J. Complementary feeding practices among infants and young children in Abu Dhabi, United Arab Emirates. *BMC Public Health* **2020**, *20*, 1308, doi:10.1186/s12889-020-09393-y.
69. Cheikh Ismail, L.; Al Dhaheri, A.S.; Ibrahim, S.; Ali, H.I.; Chokor, F.A.Z.; O'Neill, L.M.; Mohamad, M.N.; Kassis, A.; Ayesh, W.; Kharroubi, S., et al. Nutritional status and adequacy of feeding Practices in Infants and Toddlers 0-23.9 months living in the United Arab Emirates (UAE): findings from the feeding Infants and Toddlers Study (FITS) 2020. *BMC Public Health* **2022**, *22*, 319, doi:10.1186/s12889-022-12616-z.
70. Botha, M.; Basera, W.; Facey-Thomas, H.E.; Gaunt, B.; Genuneit, J.; Gray, C.L.; Kiragu, W.; Ramjith, J.; Watkins, A.; Levin, M.E. Nutrition and allergic diseases in urban and rural communities from the South African Food Allergy cohort. *Pediatr Allergy Immunol* **2019**, *30*, 511-521, doi:10.1111/pai.13058.
71. Abebe, Z.; Tariku, A.; Bikes, G.A.; Wassie, M.M.; Gonete, K.A.; Awoke, T.; Gebeye, E.; Gete, A.A.; Yesuf, M.E.; Kebede, Y., et al. Poor child complementary Feeding Practices in northwest Ethiopia: Finding from the Baseline Survey of Nutrition Project, 2016. *Ital J Pediatr* **2019**, *45*, 154, doi:10.1186/s13052-019-0747-2.
72. Fanta, M.; Cherie, H.A. Magnitude and determinants of appropriate complementary feeding practice among mothers of children age 6-23 months in Western Ethiopia. *PLoS One* **2020**, *15*, e0244277, doi:10.1371/journal.pone.0244277.

73. Ahmed, J.A.; Sadeta, K.K.; Lenbo, K.H. Magnitude and factors associated with appropriate complementary feeding practice among mothers of children 6-23 months age in Shashemene town, Oromia- Ethiopia: Community based cross sectional study. *PLoS One* **2022**, *17*, e0265716, doi:10.1371/journal.pone.0265716.
74. Esan, D.T.; Adegbilero-Iwari, O.E.; Hussaini, A.; Adetunji, A.J. Complementary feeding pattern and its determinants among mothers in selected primary health centers in the urban metropolis of Ekiti State, Nigeria. *Sci Rep* **2022**, *12*, 6252, doi:10.1038/s41598-022-10308-7.
75. Kinabo, J.; Mamiro, P.; Mwanri, A.; Bundala, N.; Kulwa, K.; Picado, J.; Ntwenya, J.; Nombo, A.; Mzimbiri, R.; Ally, F., et al. Adequacy of macro and micronutrients in infants and young children's diets in Zanzibar, Tanzania. *Afr Health Sci* **2019**, *19*, 3063-3077, doi:10.4314/ahs.v19i4.28.
76. Tran, M.M.; Lefebvre, D.L.; Dai, D.; Dharma, C.; Subbarao, P.; Lou, W.; Azad, M.B.; Becker, A.B.; Mandhane, P.J.; Turvey, S.E., et al. Timing of food introduction and development of food sensitization in a prospective birth cohort. *Pediatr Allergy Immunol* **2017**, *28*, 471-477, doi:10.1111/pai.12739.
77. Sirina, I.; Strele, I.; Siksna, I.; Gardovska, D. Eating Patterns and Food Choices of Latvian Infants during Their First Year of Life. *Medicina (Kaunas)* **2018**, *54*, 7. doi:10.3390/medicina54010007.
78. Klerks, M.; Roman, S.; Bernal, M.J.; Haro-Vicente, J.F.; Sanchez-Siles, L.M. Complementary Feeding Practices and Parental Pressure to Eat among Spanish Infants and Toddlers: A Cross-Sectional Study. *Int J Environ Res Public Health* **2021**, *18*, 1982. doi:10.3390/ijerph18041982.
79. Wu, H.H.; Zhang, Y.Q.; Zong, X.N.; Li, H. Changes of feeding patterns in Chinese city children under 2 years from 1985 to 2015: results from a series of national cross-sectional surveys. *World J Pediatr* **2019**, *15*, 176-181, doi:10.1007/s12519-018-0214-y.
80. Khor, G.L.; Lee, S.S. Complementary Foods and Milk-Based Formulas Provide Excess Protein but Suboptimal Key Micronutrients and Essential Fatty Acids in the Intakes of Infants and Toddlers from Urban Settings in Malaysia. *Nutrients* **2021**, *13*, 2354. doi:10.3390/nu13072354.
81. Pries, A.M.; Huffman, S.L.; Champeny, M.; Adhikary, I.; Benjamin, M.; Coly, A.N.; Diop, E.H.I.; Mengkheang, K.; Sy, N.Y.; Dhungel, S., et al. Consumption of commercially produced snack foods and sugar-sweetened beverages during the complementary feeding period in four African and Asian urban contexts. *Matern Child Nutr* **2017**, *13 (*Suppl 2*)*, e12412. doi:10.1111/mcn.12412.
82. Akpaki, K.; Galibois, I.; Blaney, S. Feeding practices and factors associated with the provision of iron-rich foods to children aged 6-23 months in Matam area, Senegal. *Public Health Nutr* **2021**, *24*, 4442-4453, doi:10.1017/s1368980021002718.
83. Au, L.E.; Gurzo, K.; Paolicelli, C.; Whaley, S.E.; Weinfield, N.S.; Ritchie, L.D. Diet Quality of US Infants and Toddlers 7-24 Months Old in the WIC Infant and Toddler Feeding Practices Study-2. *J Nutr* **2018**, *148*, 1786-1793, doi:10.1093/jn/nxy192.
84. Borger;, C.; Weinfield;, N.; Zimmerman;, T.; MacAllum;, C.; DeMatteis;, J.; Whaley;, S.; Ritchie;, L.; Sallack;, L.; Au;, L.; Jenkins;, F., et al. *WIC Infant and Toddler Feeding Practices Study 2 (ITFPS-2): Second Year Report.* US Department of Agriculture: Alexandria, VA, USA, 2018.
85. Masztalerz-Kozubek, D.; Zielinska, M.A.; Rust, P.; Majchrzak, D.; Hamulka, J. The Use of Added Salt and Sugar in the Diet of Polish and Austrian Toddlers. Associated Factors and Dietary Patterns, Feeding and Maternal Practices. *Int J Environ Res Public Health* **2020**, *17*, 5025. doi:10.3390/ijerph17145025.
86. Pries, A.M.; Huffman, S.L.; Adhikary, I.; Upreti, S.R.; Dhungel, S.; Champeny, M.; Zehner, E. High consumption of commercial food products among children less than 24 months of age and product promotion in Kathmandu Valley, Nepal. *Matern Child Nutr* **2016**, *12 Suppl 2*, 22-37, doi:10.1111/mcn.12267.
87. Moreira, L.C.Q.; Oliveira, E.B.E.; Lopes, L.H.K.; Bauleo, M.E.; Sarno, F. Introduction of complementary foods in infants. *Einstein (Sao Paulo)* **2019**, *17*, eAO4412, doi:10.31744/einstein_journal/2019AO4412.
88. Spaniol, A.M.; da Costa, T.H.M.; Bortolini, G.A.; Gubert, M.B. Breastfeeding reduces ultra-processed foods and sweetened beverages consumption among children under two years old. *BMC Public Health* **2020**, *20*, 330, doi:10.1186/s12889-020-8405-6.
89. Chouraqui, J.P.; Delmas, B.; Le Bris, M.; Bellaiche, M.; Jung, C.; Hanh, T. Physicians advice, parental practice and adherence to doctor's advice: an original survey on infant feeding. *BMC Pediatr* **2019**, *19*, 313, doi:10.1186/s12887-019-1697-y.
90. Scott, J.A.; Gee, G.; Devenish, G.; Ha, D.; Do, L. Determinants and Sources of Iron Intakes of Australian Toddlers: Findings from the SMILE Cohort Study. *Int J Environ Res Public Health* **2019**, *16*, 181. doi:10.3390/ijerph16020181.

91. Herrick, K.A.; Fryar, C.D.; Hamner, H.C.; Park, S.; Ogden, C.L. Added Sugars Intake among US Infants and Toddlers. *J Acad Nutr Diet* **2020**, *120*, 23-32, doi:10.1016/j.jand.2019.09.007.
92. Lopes, W.C.; Pinho, L.; Caldeira, A.P.; Lessa, A.D.C. Consumption of ultra-processed foods by children under 24 months of age and associated factors. *Rev Paul Pediatr* **2020**, *38*, e2018277, doi:10.1590/1984-0462/2020/38/2018277.
93. Cainelli, E.C.; Gondinho, B.V.C.; Palacio, D.D.C.; Oliveira, D.B.; Reis, R.A.; Cortellazzi, K.L.; Guerra, L.M.; Cavalcante, D.F.B.; Pereira, A.C.; Bulgareli, J.V. Ultra-processed foods consumption among children and associated socioeconomic and demographic factors. *Einstein (Sao Paulo)* **2021**, *19*, eAO5554, doi:10.31744/einstein_journal/2021AO5554.
94. Soares, M.M.; Juvanhol, L.L.; Ribeiro, S.A.V.; Franceschini, S.; Araújo, R.M.A. Prevalence of processed and ultra-processed food intake in Brazilian children (6-24 months) is associated with maternal consumption and breastfeeding practices. *Int J Food Sci Nutr* **2021**, *72*, 978-988, doi:10.1080/09637486.2021.1885016.
95. Spaniol, A.M.; da Costa, T.H.M.; Souza, A.M.; Gubert, M.B. Early consumption of ultra-processed foods among children under 2 years old in Brazil. *Public Health Nutr* **2021**, *24*, 3341-3351, doi:10.1017/s1368980020004759.
96. Giesta, J.M.; Zoche, E.; Corrêa, R.D.S.; Bosa, V.L. Associated factors with early introduction of ultra-processed foods in feeding of children under two years old. *Cien Saude Colet* **2019**, *24*, 2387-2397, doi:10.1590/1413-81232018247.24162017.
97. Laterra, A.; Ayoya, M.A.; Beaulière, J.M.; Bienfait, M.; Pachón, H. Infant and young child feeding in four departments in Haiti: mixed-method study on prevalence of recommended practices and related attitudes, beliefs, and other determinants. *Rev Panam Salud Publica* **2014**, *36*, 306-313.
98. Afeiche, M.C.; Villalpando-Carrión, S.; Reidy, K.C.; Fries, L.R.; Eldridge, A.L. Many Infants and Young Children Are Not Compliant with Mexican and International Complementary Feeding Recommendations for Milk and Other Beverages. *Nutrients* **2018**, *10*, 466. doi:10.3390/nu10040466.
99. Bournez, M.; Ksiazek, E.; Charles, M.A.; Lioret, S.; Brindisi, M.C.; de Lauzon-Guillain, B.; Nicklaus, S. Frequency of Use of Added Sugar, Salt, and Fat in Infant Foods up to 10 Months in the Nationwide ELFE Cohort Study: Associated Infant Feeding and Caregiving Practices. *Nutrients* **2019**, *11*, doi:10.3390/nu11040733.
100. Green, M.; Hadihardjono, D.N.; Pries, A.M.; Izwardy, D.; Zehner, E.; Huffman, S.L. High proportions of children under 3 years of age consume commercially produced snack foods and sugar-sweetened beverages in Bandung City, Indonesia. *Matern Child Nutr* **2019**, *15 Suppl 4*, e12764, doi:10.1111/mcn.12764.
